# Effectiveness of home-based records on maternal, newborn and child health outcomes: A systematic review and meta-analysis

**DOI:** 10.1371/journal.pone.0209278

**Published:** 2019-01-02

**Authors:** Olivia Magwood, Victoire Kpadé, Kednapa Thavorn, Sandy Oliver, Alain D. Mayhew, Kevin Pottie

**Affiliations:** 1 C.T. Lamont Primary Health Care Research Centre, Bruyère Research Institute, Ottawa, ON, Canada; 2 Ottawa Hospital Research Institute, Ottawa, ON, Canada; 3 School of Epidemiology and Public Health, University of Ottawa, Ottawa, ON, Canada; 4 Institute for Clinical and Evaluative Sciences, Ottawa, ON, Canada; 5 Department of Social Science, UCL Institute of Education, London, United Kingdom; 6 University of Johannesburg, Johannesburg, South Africa; 7 Department of Family Medicine, School of Epidemiology and Community Medicine, University of Ottawa, Ottawa, ON, Canada; University of South Florida, UNITED STATES

## Abstract

Home-based records (HBRs) may improve the health of pregnant women, new mothers and their children, and support health care systems. We assessed the effectiveness of HBRs on maternal, newborn and child health reporting, care seeking and self-care practice, mortality, morbidity and women’s empowerment in low-, middle- and high-income countries. We conducted a systematic search in MEDLINE, EMBASE, CENTRAL, Health Systems Evidence, CINAHL, HTA database, NHS EED, and DARE from 1950 to 2017. We also searched the WHO, CDC, ECDC, JICA and UNAIDS. We included randomised controlled trials, prospective controlled trials, and cost-effectiveness studies. We used the Cochrane Risk of Bias tool to appraise studies. We extracted and analyzed data for outcomes including maternal, newborn and child health, and women’s empowerment. We synthesized and presented data using GRADE Evidence Profiles. We included 14 studies out of 16,419 identified articles. HBRs improved antenatal care and reduced likelihood of pregnancy complications; improved patient–provider communication and enhanced women’s feelings of control and empowerment; and improved rates of vaccination among children (OR: 2·39, 95% CI: 1.45–3·92) and mothers (OR 1·98 95% CI:1·29–3·04). A three-year follow-up shows that HBRs reduced risk of cognitive delay in children (p = 0.007). HBRs used during the life cycle of women and children in Indonesia showed benefits for continuity of care. There were no significant effects on healthy pregnancy behaviors such as smoking and consumption of alcohol during pregnancy. There were no statistically significant effects on newborn health outcomes. We did not identify any formal studies on cost or economic evaluation. HBRs show modest but important health effects for women and children. These effects with minimal-to-no harms, multiplied across a population, could play an important role in reducing health inequities in maternal, newborn, and child health.

## Introduction

Home-based records (HBRs) are used in over 163 countries or territories [1]. These records are paper or electronic documents that pregnant women and caregivers commonly maintain and use in the household to monitor the health of the household’s children. The contents of HBRs cover one or more components of preventive or curative antenatal, postnatal, newborn, and child health, including vaccination and nutrition. These records may improve maternal, newborn and child health and development in both developed and developing countries [2–4]. Demographic health surveys from 1993 to 2013 indicate prevalence of country-specific HBR usage was at least 90% in all regions around the world, except for South-East Asia, where prevalence was estimated to be 84% [5]. However, use of HBRs is inconsistent across and within countries; and despite the benefits shown in primary studies, parents and health care practitioners often underutilize HBRs or use them inappropriately [1, 3].

In 2015, the number of maternal deaths due to preventable pregnancy- or childbirth-related complications was 303,000 [6], of which 99% occurred in settings of limited resources [7]. In 2016, 5.6 million children under age five died from preventable causes [8]. Most of these deaths occurred in low- and middle-income countries (LMICs) [8]. The UN Sustainable Development Goal 3 calls for an end to the preventable deaths of children under age five, by 2030 [9].

HBRs are a simple, globally applicable intervention that may improve the health and lives of women and children. HBRs have a unique role in linking mothers and caregivers to maternal, newborn and child health information and health care. In addition, health education messages are often included in these records so as to promote better health care seeking, healthy behaviours, and safe home care practices. HBRs come in different forms, starting with the most basic antenatal or vaccination-only cards, and progressing to vaccination-plus cards, maternal and child health books, and electronic records. Electronic records provide patients with access to their health information through the internet, cellular devices, and tablets. The growth of electronic HBRs reflects the increasing trend in the digitization of health care [10].

In 1994, World Health Organization (WHO) recommended that all women of childbearing age should have home-based maternal records [11]. More recently, the WHO has developed guidance to improve the use and design of HBRs for immunisation [12]. Additionally, the WHO’s health systems interventions to improve the utilisation and quality of antenatal care include women-held case notes [13]. Although, in 1992, a WHO collaborative study [2] evaluated the process and functioning of HBRs for maternal health in eight countries, global evidence on their effects on maternal, newborn and child health has never been systematically reviewed, nor has a global assessment of the benefits of using different types of HBRs been conducted [14]. The objective of this review is to synthesise and compare the evidence of the health and cost effectiveness of HBRs for improving maternal, newborn and child health outcomes, including empowerment outcomes for women. This study also aims to determine whether particular types of HBRs improve these outcomes more than others.

## Methods

### Search strategy and selection criteria

We followed the PRISMA reporting guidelines [15] for the design and reporting of this systematic review and meta-analysis. This study addresses women and other caregivers, and practitioners (P), home-based records (I), versus limited or no use of home-based records (C) using international consensus outcomes (O). We used the GRADE approach to systematically estimate the certainty of the evidence for each outcome (Table 1) [16]. We published a protocol on the Cochrane Equity Methods website [17].

**Table 1:** Certainty of evidence and definitions.

Certainty rating	Definition
High	Further research is very unlikely to change our confidence in the estimate of effect
Moderate	Further research is likely to have an important impact on our confidence in the estimate of effect and may change the estimate
Low	Further research is very likely to have an important impact on our confidence in the estimate of effect and is likely to change the estimate
Very low	Any estimate of effect is very uncertain

### Research questions

1. For women during pregnancy and after birth, and for newborns, children and caregivers [P], does use of any home-based records [I], compared with no use of any home-based records [C], improve maternal, newborn and child health outcomes [O]?
2. For women during pregnancy and after birth, and for newborns, children and caregivers [P], does use of any home-based records [I], compared with inconsistent use (low use) of any home-based records [C], improve maternal, newborn and child health outcomes [O]?
3. For women during pregnancy and after birth, and for newborns, children and caregivers [P], does use of different types of home-based records [I], improve maternal, newborn and child health outcomes [O]?
4. For women during pregnancy and after birth, and for caregivers [P], does any use of home-based records [I], compared with no use of any home-based records [C], improve health service outcomes [O]?
5. For women during pregnancy and after birth, and for caregivers [P], does any use of home-based records [I], compared with inconsistent use (low use) of any home-based records [C], improve health service outcomes [O]?
6. For women during pregnancy and after birth, and for caregivers [P], does use of different types of home-based records [I] improve health service outcomes [O]?
7. What is the cost-effectiveness and what are the resource requirements for HBRs?

We searched for randomised controlled trials (RCTs), including cluster RCTs, controlled trials, and interrupted time-series (ITS) studies. We also searched for cost and economic evaluation studies. A health sciences librarian and health economist (KT) developed the search strategy. We searched the following electronic databases: MEDLINE, Cochrane Central Register of Controlled Trials (CENTRAL), EMBASE, Health Systems Evidence, Cumulative Index to Nursing and Allied Health Literature (CINAHL), HTA database, NHS EED, and DARE. We applied no date or language restrictions. We used a combination of indexed terms and free-text words (S1 File). We considered including the primary studies of relevant systematic reviews that came up in our search. If more than one version of a study was identified, we selected the most recent version. If the two versions reported on different outcomes, then both studies were included. We also searched grey literature from the US Centre for Disease Control and Prevention, the European Centre for Prevention and Disease Control, JICA, UNAIDS, and the WHO. For medical economics, this also included the Canadian Agency for Drugs and Technologies in Health, the Institute of Health Economics, the National Institute for Health and Care Excellence, EuroScan, and the database of the Centre for Reviews and Dissemination. We uploaded the search records to a reference-managing software package to facilitate the study-selection process.

We included studies for which we could retrieve the full texts that included pregnant women, mothers, or children under 10 years of age [18, 19]. The intervention of interest was any form of a patient-held HBR that had impacts on maternal, newborn or child health outcomes. Patient diaries, provider-held records, and mobile health interventions that involved text messages were not considered eligible interventions. Review authors (OM, VK, KT) independently assessed for inclusion the potential studies the search strategy identified. Any disagreements were resolved through discussion or, if required, by consulting a third reviewer (KP). If required, study authors were contacted, and articles were translated.

### Data analysis

We developed a standardised data-extraction sheet which included the study design, population, intervention, comparison, outcomes, results, conclusions, and funding sources. Two reviewers (OM, VK) independently extracted the data in duplicate. They compared the results and resolved disagreements through discussion. We assessed the methodological quality of the RCTs and controlled trials, using the Cochrane Risk of Bias Tool [20]. We also planned to assess any ITS studies, using the EPOC criteria [21]. By default, controlled trials were judged as “high risk of bias” for randomisation and allocation concealment. The methodological quality of the included economic evaluation studies was assessed using the Drummond checklist [22]. The certainty of the evidence for the effects on the study group was assessed using GRADE methodology [23].

Where possible, results were meta-analysed using RevMan 5 software [24] using a random-effects model and summary effects are given as odds ratios or relative risks. When possible, we pooled direct and indirect estimates in a network meta-analysis to produce the estimates on important patient outcomes of the relative effects of each record design.

## Results

**Fig 1:**
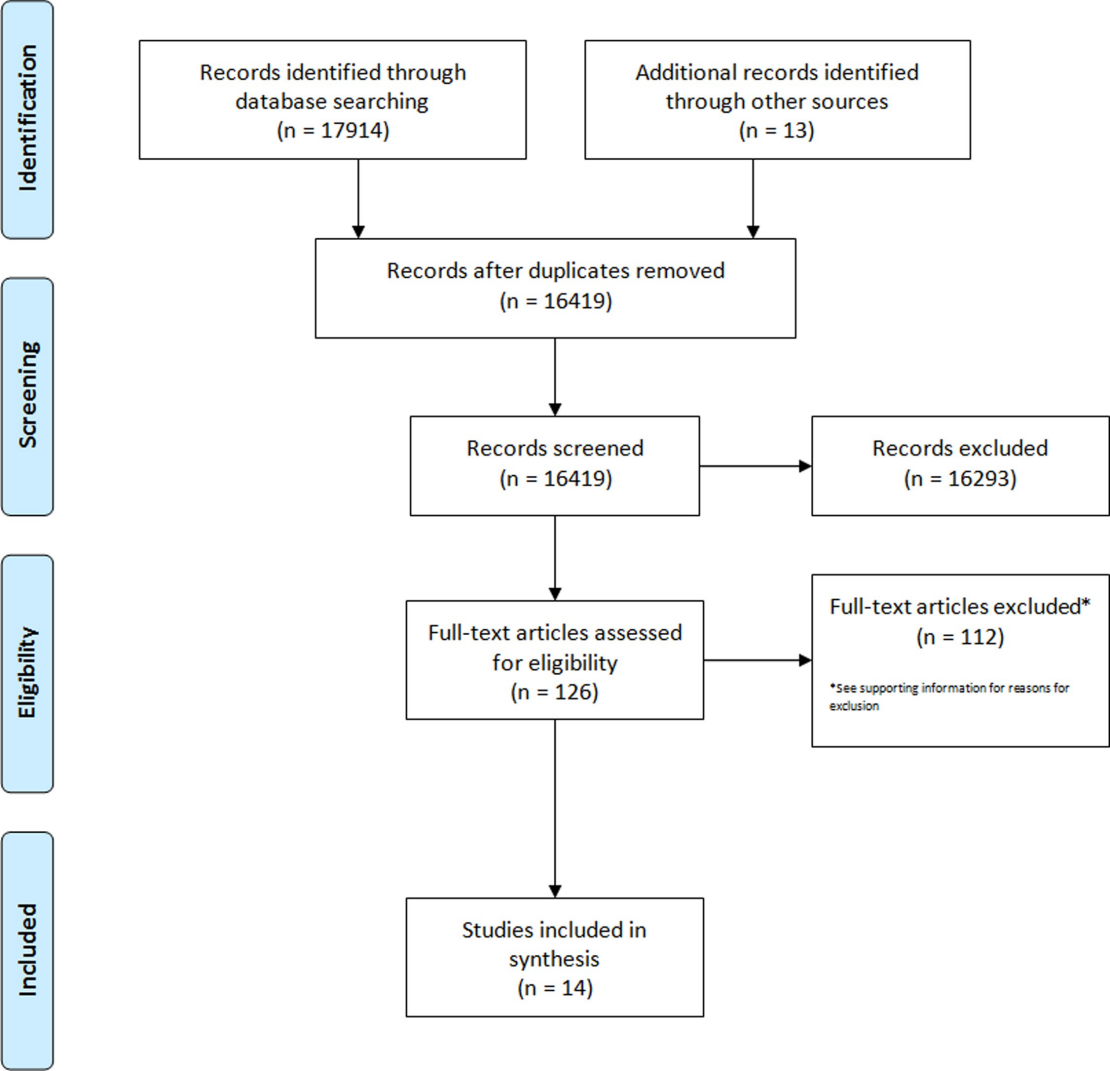
PRISMA flow diagram.

We screened 16,419 titles and abstracts for eligibility on the effectiveness and cost-effectiveness of home-based records. In all, 14 studies met our inclusion criteria (Fig 1). Studies were excluded at the full text stage due to irrelevant population (n = 2), full text could not be retrieved (n = 1), and study design or irrelevant intervention (n = 109) (S2 File).

**Fig 2:**
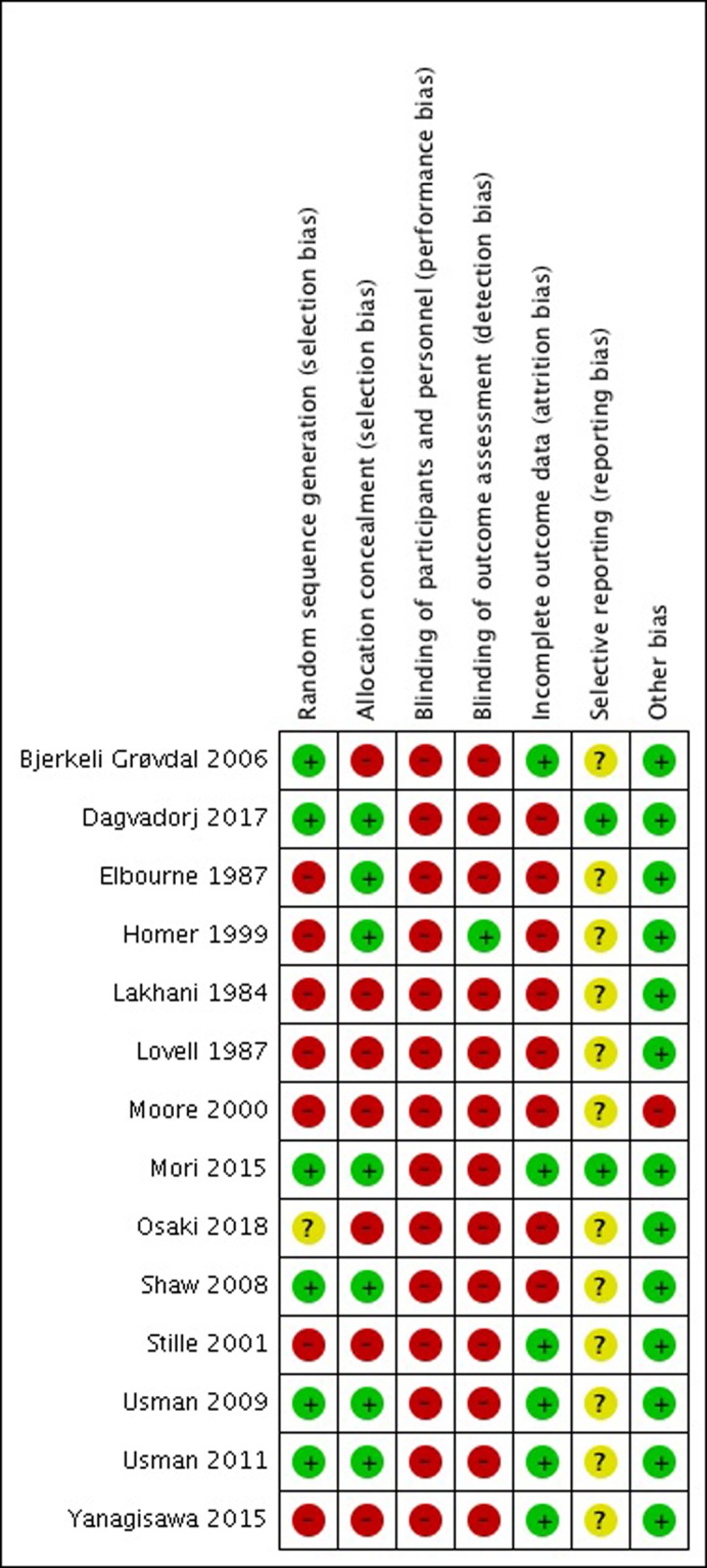
Risk of Bias summary. Review authors' judgements (Low, Unclear and High) about each risk of bias item for each individual study using the Cochrane Risk of Bias tool.

Among the included studies were 9 RCTs, 1 cluster RCT and a three-year follow-up, and 2 controlled trials. We did not find any eligible ITS studies. Studies were conducted in high-income countries: Australia (n = 1), England (n = 4), Norway (n = 1), United States of America (USA) (n = 1); and in low- and middle-income countries: Cambodia (n = 1), Indonesia (n = 1), Mongolia (n = 1) and Pakistan (n = 2). Table 2 shows the intervention and population descriptions; Fig 2 shows a summary of the risk of bias.

**Table 2:** Characteristics of included studies.

	Study design	Study setting	Study population	Sample size	Intervention	Comparator	Outcomes of interest
Bjerkeli Grøvdal et al. 2006	RCT	10 municipalities in More and Romsdal county in Norway with 14 child health centers, both urban and rural.	Children six weeks to five years old born between 1 August 2000 and 1 October 2001.	309	Parent-held child health record (including appointments, vaccinations, steps in child development, room for both health personnel and parents to make notes) in addition to the ordinary national health surveillance program.	Ordinary national health surveillance program	Parent-professional collaboration, healthcare utilization, and parents’ knowledge about child health matters and illness
Elbourne et al. 1987	RCT	Peripheral consultant clinic. Newbury, West Berkshire, England.	Women less than 34 weeks gestation who booked for antenatal care with E.H. at the peripheral clinic at the Sandleford Hospital, Newbury between 1 January and 30 June 1984.	247	Woman-held obstetric records	Co-operation card (abbreviated version of the full obstetric record)	Patient satisfaction, patient control, communication with health providers, number of antenatal classes, smoking, duration of labor, baby's father's involvement, breastfeeding, likelihood of depression, use of analgesia, and savings of clerical resources
Homer et al. 1999	RCT	Australian metropolitan maternity service	English-speaking women attending the hospital clinic for their first antenatal visit between January to December 1997.	126	Woman-held antenatal record	Co-operation card (abbreviation of the full antenatal record)	Feeling of control and women's satisfaction during pregnancy
Lakhani et al. 1984	Randomized controlled evaluation	St Thomas's Hospital, London, England.	Mothers resident in the West Lambeth Health District and discharged from obstetric wards at St Thomas's hospital between May and August 1980.	299	Home-based child health record booklet. The booklet contained spaces for a photograph; name; birth statistics; four address changes; important names and telephone numbers, developmental mile-stones; and a schedule and record of immunization and developmental assessment clinic attendance. Two single sex weight charts, from 0 to 2 years and 2 to 10 years were included.	No intervention for the period of May to August 1980. In September 1980, it had been decided that booklets would be distributed to all mothers in the district.	Relationship between parents and professionals, communication between professionals, health education, and continuity of care
Lovell et al. 1987	RCT	Antenatal clinic in St. Thomas’s Hospital, London, England.	Mothers attending the antenatal clinic between 20 June to 7 November 1984.	228	Woman-held maternity case notes	Co-operation card (abbreviated version of full record). Mothers in this group handled their full maternity notes while waiting to be seen in the antenatal clinic and thus had access to their contents during this time.	Satisfaction with the care given; their sense of control and self-confidence; their communication with staff; and the involvement of the babies' fathers, health-related behavior including attendance at antenatal appointments, breast-feeding, smoking and the consumption of alcohol, practical problems or detectable adverse clinical outcomes.
Moore et al. 2000	Randomized controlled two-phase trial.	Education Department within Leicestershire county, England.	Children with severe and obvious disabilities referred from the local Education Department	71	Personal child health record for children with a disability, as a supplement to the Leicestershire child health record. New section included pages for recording contact info of child's healthcare professionals, space for child's medical diagnoses and medications, appointment logs, diary of achievements, info about organizations relevant to children with disabilities	No intervention (usual care)	Usability, value, perception of health care received, communication between the family and healthcare professional
Mori et al. 2015 3 year follow up: Dagvadorj et al. 2017	Cluster randomized controlled trial	18 units (16 *soums* and 2 *bags*) in the Bulgan Province of Mongolia.	Pregnant women and their infants living in the Bulgan province of Mongolia between May 2009 and September 2010.	501	Maternal and Child Health handbook containing a log for recording information on maternal health and personal information, course of pregnancy, delivery and postpartum health, weight during and after pregnancy, dental health, parenting classes, child development milestones from the ages of 0–6 years, immunization and illnesses, and height and weight charts for children.	No intervention for the period of the original trial. In 2010 the MCH handbook was implemented as part of the national health policy.	Antenatal clinic attendance, health seeking behaviors, client-provider communication, maternal physical and mental health, neonatal health and healthy behaviors
				386			Risk of cognitive developmental delay
Osaki et al. 2018	Cluster randomized controlled trial	13 health centers in Garut district of rural Java, Indonesia.	Pregnant women attending one of the selected health centers between 2007 and 2009.	454	Maternal and Child Health Handbook documenting and monitoring services provided and a point-of-care information resource.	No intervention (usual care)	Maternal immunization, antenatal clinic appointments, vitamin A intake, feeding practices, child growth and development.
Shaw et al. 2008	RCT	Maternity Centre of Hamilton, Ontario, Canada.	Women presenting to a primary care maternity center before 28 week’s gestation between September 2004 and January 2006.	106	Access to a website with pregnancy health information and access to their own antenatal health record, through a condensed version of the clinic’s antenatal care planner. This planner contained information and reminders generated by the presence of any of the following risk factors: being a current smoker, having a history of premature labor, having a history of previous Caesarean section, being Rh negative, or being over 35 years of age at the time of delivery. The website with pregnancy health information gave information on labor and delivery, integrated prenatal screening, nutrition, smoking cessation, local community resources, tests during pregnancy, sexuality, and postpartum contraception.	Access to website with pregnancy information only, giving information on labor and delivery, integrated prenatal screening, nutrition, smoking cessation, local community resources, tests during pregnancy, sexuality, and postpartum contraception.	Frequency of use, satisfaction with and perceived usefulness of web-based information
Stille et al. 2001	Controlled trial	3 inner-city pediatric primary care sites in Hart- ford, Connecticut, USA.	Infants born in any hospital in Hartford between October 1997 and May 1998 who presented for their first well-child visits and 1 or the 3 sites under 28 days of age, whose primary caregiver spoke English or Spanish.	315	Routine information about immunizations and received a 2- component intervention. 1) Interactive graphic card containing: list of immunizations in the infant's primary series, with a space where stickers could be placed for each immunization, and a space where parents could put their child's picture. Information targeted to remedy specific deficiencies in parent knowledge, about immunization timing, safety, and contraindications. 2) Explanation of the card by the provider in the provider's own words and answering of any caregiver questions.	Routine information about immunizations	Immunization status of 3-dose DTP vaccination.
Usman et al. 2009	RCT	EPI centers in urban areas of Karachi city, Pakistan.	Children visiting the selected EPI centers for DTP1 and residing in the same area for the last 6 months.	1461	Redesigned immunization card and/or center-based education session. The redesigned card was a new and simpler immunization card whose most important function was to act as a constant reminder to mothers for next immunization visit. The larger immunization card (15.5cm×11.5cm when folded) showed only the next immunization date and day on both outer sides in Microsoft Word font 42. The remainder of the information such as name of the EPI center, card number, card’s date of issue, child’s name and address, complete immunization schedule dates, and instructions and information for the mother was written on the inner sides of the folded card. The center-based education was a 2–3 min conversation with mother to convey the importance of the completion of immunization schedule and to explain the potential adverse impact of incomplete immunization on child’s health.	Old EPI immunization card. It is small (9 x 8.5 cm, when folded); hence, the information on child’s identity, immunization schedule, information for mothers and next immunization visit dates is crowded together and appears disorderly. And the next immunization date is hand-written by the EPI staff, often in very small and irregular letters.	Immunization status of 3-dose DTP vaccination.
Usman et al. 2011	RCT	6 EPI centers in the rural peripheries of Karachi, Pakistan.	Children visiting the selected EPI centers for DTP1 and residing in the same area for the last 6 months.	1506	Redesigned immunization card and/or center-based education session. The redesigned card was a new and simpler immunization card whose most important function was to act as a constant reminder to mothers for next immunization visit. The larger immunization card (15.5cm×11.5cm when folded) showed only the next immunization date and day on both outer sides in Microsoft Word font 42. The remainder of the information such as name of the EPI center, card number, card’s date of issue, child’s name and address, complete immunization schedule dates, and instructions and information for the mother was written on the inner sides of the folded card. The center-based education was a 2–3 min conversation with mother to convey the importance of the completion of immunization schedule and to explain the potential adverse impact of incomplete immunization on child’s health.	Old EPI immunization card. It is small (9 x 8.5 cm, when folded); hence, the information on child’s identity, immunization schedule, information for mothers and next immunization visit dates is crowded together and appears disorderly. And the next immunization date is hand-written by the EPI staff, often in very small and irregular letters.	Immunization status of 3-dose DTP vaccination.
Yanagisawa et al. 2015	Pre-post intervention controlled trial	Health centers located in Kampong Cham Province, Cambodia.	Women who had given birth 1 year prior to the survey living in an intervention or control area.	640	Maternal and Child Health Handbook	Standard Cambodian Child Health Card (child growth card) and Mother Health Record. Also received the tetanus immunization card and the vitamin A intake record	Maternal behaviours including antenatal attendance, deliveries attended by SBAs and deliveries at health facilities). Secondary outcomes included maternal knowledge of danger signs during pregnancy and delivery, prevention of anaemia, prevention of intestinal parasites, mother-to-child HIV transmission, early breastfeeding practice and child immunization

No RCTs or controlled trials reported on maternal mortality, pregnancy nutrition, the number of postpartum visits, care seeking for postpartum complications, postpartum family planning, growth monitoring, development monitoring, continued breastfeeding and warmth and hygiene of the newborn. We did not identify any evidence comparing different types and designs of HBRs, and therefore results are reported considering HBRs as a single intervention. A complete summary of outcomes, effect sizes and certainty of evidence is provided in GRADE evidence profiles (S3 File).

### Maternal health outcomes

HBRs had positive effects on some maternal health outcomes. In Mongolia and Indonesia, the Maternal and Child Health (MCH) handbook, compared with no HBR, significantly increased the proportion of women who had six or more antenatal clinic attendances (OR: 1·93, 95% CI: 1·48–2·53) [25, 26]. In Mongolia, clinical complications in pregnancy, as listed by Mongolia’s Department of Health, were more easily identified in pregnant women with a HBR (OR: 2·33, 95% CI: 1·21–4·51) [25]. However, there were no effects of HBRs on the clinical outcomes of mothers when compared to abbreviated coop cards (OR: 0·63, 95% CI: 0·37–1·1) [27]. In Indonesia, women in areas where the MCH handbook is used were more likely to receive two doses of tetanus immunisation (OR 1·98 95% CI:1·29–3·04) [26]. An RCT also demonstrates that HBRs increase women’s feelings of control during antenatal care (OR: 1·45, 95% CI: 1·08–1·95) [28]. Findings suggest that HBRs had no effect on smoking (RR 1·01, 95% CI: 0·9–1·04) or alcohol consumption (RR: 1·07, 95% CI: 0·97–1·18) during pregnancy [25]. Similarly, when comparing women held maternity cards to abbreviated versions, two RCTs showed that there was no effect on healthy pregnancy behaviour [27, 28] (GRADE Certainty of evidence: low to very low). However, in households where the woman carried the MCH handbook, one RCT reported a decrease in smoking among family members living in the same household (RR 0·84, 95% CI:0·7–0·99) [25] (GRADE Certainty of evidence: Low).

### Newborn health outcomes

Findings suggest no statistical effects of HBRs on newborn outcomes. In Mongolia, MCH handbooks had no effects compared to the unspecified pre-existing system in the control group on neonatal death or stillbirths (RR 1·0 95% CI: 0·99–1.01, p = 0·512) [25]. In the United Kingdom, full pregnancy case notes had no effect, compared to a briefer ‘co-op card’, on neonatal death or stillbirths (RR 1·04 95% CI: 0·15–1·21) [27] (GRADE certainty of evidence: very low). Additionally, there was no effect on immediate breastfeeding in Mongolia (RR 1.07 95CI: 0.97–1.18, GRADE certainty of evidence: Moderate) [25] or in the United Kingdom (OR 1.09 95CI: 0.56–2.11, GRADE certainty of evidence: very low) [27].

### Child health outcomes

**Fig 3:**
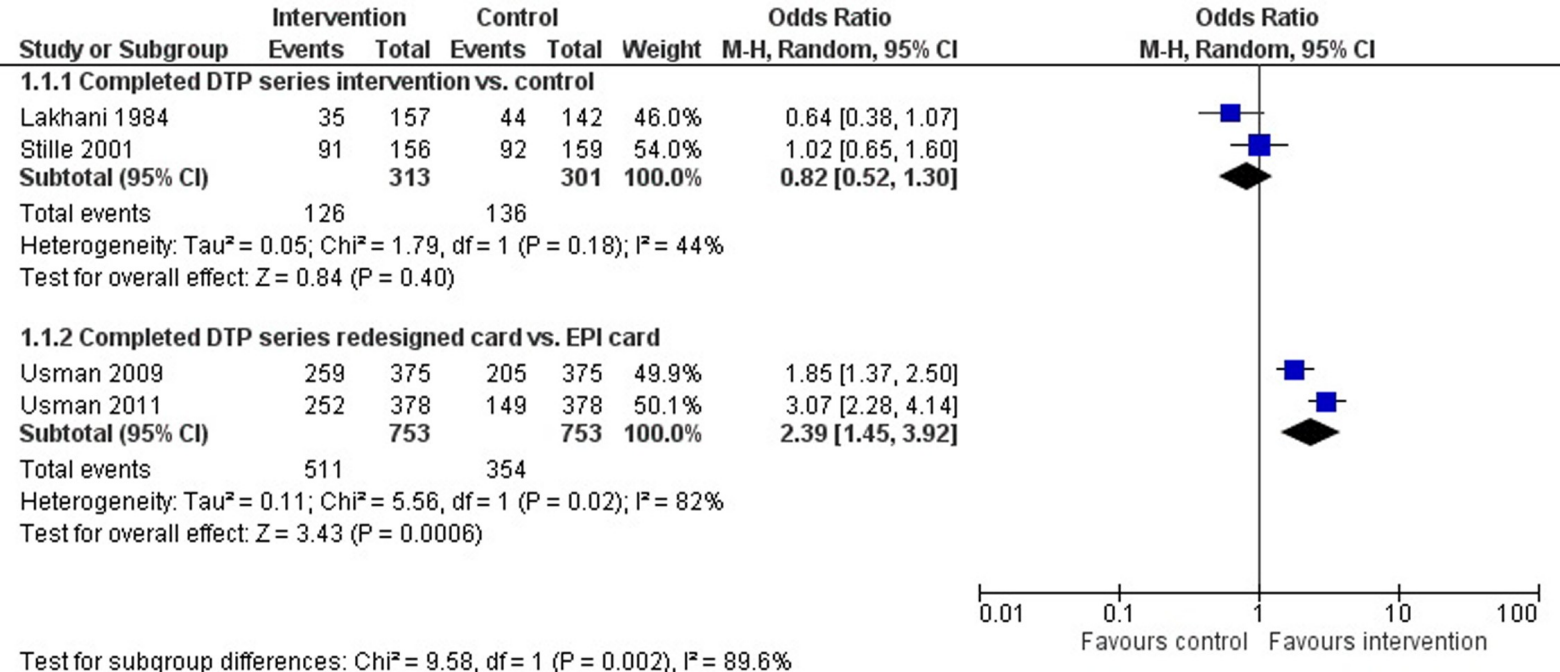
Meta-analysis of childhood vaccination (DTP) series completion among individuals using HBRs as compared to no HBR (1.1.1) or existing EPI cards (1.1.2).

Among child health outcomes, evidence indicates that HBRs may have an impact on immunisation rates, growth and development. Age-appropriate immunisation, including a three-dose series of diphtheria-pertussis-tetanus (DPT) by seven months of age, improved with newly designed immunisation cards and educational interventions (Fig 3). In Pakistan, studies completed in both rural and urban areas show that using a redesigned immunisation card results in a significant improvement in immunisation uptake, compared to a standard expanded program on immunisation (EPI) card (OR: 2·39, 95% CI: 1·45–3·92) (GRADE certainty of evidence: moderate) [29, 30]. In Indonesia, after using the MCH handbook, there were fewer underweight children (OR 0·33, 95% CI 0·12–0·94; GRADE certainty of evidence: very low) and fewer children with stunted growth (OR 0·53, 95% CI 0·30–0·92; GRADE certainty of evidence: low), after adjusting for maternal BMI and child birth weight [26]. In Mongolia, a 3-year follow-up showed a reduced risk of cognitive development delay in children (OR 0·32, 95% CI:0·14–0·73, p-value = 0.007) (GRADE certainty of evidence: very low) [31]. Similarly, in Indonesia, the use of the MCH handbook is shown to influence husbands’ involvement in providing their child with developmental stimulation (OR 1·62 95% CI:1·06–2·48).

Respondents using the MCH handbook in Indonesia were more concerned about continuity of care throughout the maternal, newborn and child period. They were more likely to receive multiple services, including two doses of tetanus immunisation, antenatal care four times, professional assistance during child delivery, ensure that their child took vitamin A supplements, exclusively breastfeed during infancy, and begin complementary feeding in 6–9 months (OR 7·13, 95% CI: 2·43–20·90; GRADE certainty of evidence: low) [26]. After a two year follow up, both intervention and control groups saw improvements in bringing their HBRs to more than two facilities, on more than two occasions, or filled in by more than two health personnel (GRADE certainty of evidence: very low) [26].

### Cost-effectiveness

We did not identify any formal studies on cost or economic evaluation. We found two articles that discuss the resource or economic implications of home-based records. One study [32] reported the results from a survey, of 195 countries, on the impact of home-based vaccination records on national immunisation programs. The survey revealed that the printing cost per record ranged from US$0.01 to US$3.36 and substantially varied across LMICs. Brown [5] shows that home-based vaccination records could lead to potential cost-savings of from US$500,000 to US$100,000; these estimates were based on an assumption that the use of the HBR could reduce re-vaccination by 20%. None of these articles qualified for inclusion as cost or economic evaluations (S2 File).

## Discussion

HBRs show modest impacts on maternal and child health outcomes, including antenatal clinic attendance, improving childhood immunisation rates, and promote feelings of control and empowerment among women. While several studies report on the prevalence of different kinds of HBRs between countries, the design, use and complexity of these records vary [3, 5]. Countries have identified the need for a standardised HBR so as to improve data transferability and secure the accuracy of data recording and transcription [33]. With a standardised design and proper utilisation between different health providers, these records could promote continuous maternal and child health care [34]. Since each country has its own challenges in delivering health care, different regional, social, and economic factors affect their ability to deliver proper health services to women and children. Organisations have highlighted the need for stakeholder engagement in the design, distribution, and implementation of HBRs for maternal and child health, so as to ensure the successful uptake and sustainability of these records [35, 36]. Additionally, there is no high quality evidence available that compares electronic versus paper HBRs for maternal and child health. Further research, here, is necessary. Finally, there is a need for more research that compares integrated HBRs (e.g., MCH Handbooks) and stand-alone records (e.g., vaccination cards, growth charts).

This systematic review provides insights into the effectiveness of HBRs on maternal, newborn and child health outcomes. High quality evidence indicates that HBRs may facilitate the identification of pregnancy complications [25]. Two RCTs show no effect on the reduction of neonatal mortality. However, findings from Japan suggest that HBRs may have played an important role in reducing this country’s infant mortality rate to one of the lowest in the world [37]. In Vietnam, MCH handbook usage shows similar decreases in the mortality rates of mothers during and shortly after pregnancy and of children under age five [4]. In Mongolia, it is also shown that HBRs have the potential to reduce child morbidity, as a three-year follow-up shows their protective effect on child cognitive development. This clinically important effect may have come from the frequent use of the part of the home-based record that increases mothers’ awareness of their children's developmental milestones and enhances their efforts to interact with their children [26, 31].

Home-based records help pregnant women remember antenatal care visits, thereby preventing missed appointments, and increasing their number of antenatal visits [25–27, 38, 39]. Integrated home-based records that are implemented in other parts of the world, such as Burundi, have also helped health practitioners provide information on prenatal care, which has increased the number of prenatal care visits [40]. This is consistent with other studies in LMICs that report on the role of HBRs in increasing health service uptake and promoting patient health behaviours [34, 41–45]. Home-based records have no effect on improving health behaviours, such as smoking and alcohol consumption. However, it is important to note the almost three decades that separate these two RCTs; the opposing results of these studies may come from the improved knowledge, over time, of the impacts of smoking and drinking behaviours during pregnancy. Additionally, while the control mothers in the Lovell (1984) study were not holding their case notes during their health care visits, they did have access to them while they were waiting in the antenatal clinic and may have benefitted from access to this information.

The use of home-based records is recommended in resource-limited settings [31]. These records improve the relationship and communication between mothers and health care providers; they also enhance women’s feelings of empowerment and promote a more efficient use of health service resources [25, 28, 38, 46]. In Palestine, women who were part of a national program noted that it is easier to ask questions about their health when they are holding their own handbook; also, women with less education became more familiar with their maternal and child health information through the guidance of healthcare providers who use these home-based records [34]. Personalised guidance, such as face-to-face interactions or maternal- and child-health-related social events, is essential for mothers who are less literate and who are using home-based records; this ensures they understand the information on pregnancy and it improves their pregnancy-related behaviours [43]. However, these benefits depend on whether all parties are using the record. Contrary to popular belief, women do not misplace their records more often than hospitals do, and the added information these records provide does not cause users to become more anxious [47]. These results have implications for hospital administrators and health planners, and it is necessary to train health care providers on the appropriate use of these records [26, 31].

The use of HBRs, including information on vaccination and the implementation of redesigned immunisation records, increases immunisation uptake in children and mothers [25, 28, 29, 37, 38]. The redesigned immunisation card has led to a higher increase in DPT series completion in children in rural areas compared to those in urban areas [28, 29]. This suggests that this type of intervention is beneficial in areas of lower literacy and in rural areas [28]. In Ethiopia and Madagascar, redesigned immunisation cards have been implemented with success and have also served as examples for Ghana, Liberia, and Myanmar [34].

The goal of this review was to evaluate the effectiveness of HBRs on maternal and child health in order to determine the role these records play in improving health outcomes. Our review highlights key gaps in the economic implications of HBRs. A few studies [5, 32] show that the costs associated with HBRs are minimal; also, the quality of these studies’ methodology was poor. Studies on the cost of illness or cost-description, although important, do not address the potential downstream benefits of HBRs and the impact on vaccine-preventable diseases. To inform decisions on resource allocation, future studies should assess the cost-effectiveness of HBRs by comparing the cost of their implementation, including infrastructure, materials, supplies, and delivery, against their health and economic benefits.

## Strengths and limitations

We used high quality methods to synthesise randomised controlled trials and controlled trials, conducted the first meta-analyses in this area, and used GRADE methods to assess the certainty of the effects. Limitations include a broad range of outcomes and, thus, too few studies available for meta-analyses. The number of studies was not sufficient to conduct subgroup analyses on various regions or low-income countries. Blinding for the intervention of the HBR was not feasible. There was heterogeneity in the interventions, particularly between integrated and non-integrated (card-type) HBRs. Most studies on electronic HBRs did not evaluate the effects of the electronic record, suggesting that more research is needed as electronic records begin to emerge. The available evidence was insufficient to use network meta-analysis to answer the question of the relative advantages of the different types of home-based records. In our systematic review, the studies did not use placebo designs and, instead, used several different interventions/comparisons. However, there was considerable heterogeneity in the outcome measures and this prevented a pooling of the effects.

## Conclusions

HBRs show modest impacts on maternal and child health outcomes: improving antenatal clinic attendance, improving childhood immunisation rates and promoting feelings of control and empowerment among women. Electronic HBRs have begun to emerge as promising tools, but evidence gaps remain. Cost effectiveness studies are also needed for both electronic and paper HBRs.

## Supporting information

S1 File Example search strategy. (PDF)

S2 File Table of excluded studies. (PDF)

S3 File GRADE evidence profiles. (PDF)

S4 File Home-based records review protocol. (PDF)

S5 File PRISMA 2009 checklist. (DOC)
