# Correction: Effectiveness of home-based records on maternal, newborn and child health outcomes: A systematic review and meta-analysis

**DOI:** 10.1371/journal.pone.0212698

**Published:** 2019-02-14

**Authors:** Olivia Magwood, Victoire Kpadé, Kednapa Thavorn, Sandy Oliver, Alain D. Mayhew, Kevin Pottie

There are errors in the Funding section. The correct funding information is as follows: This research was supported by the World Health Organization through a grant received from the Japan International Cooperation Agency (JICA). The World Health Organization was actively involved in study design, data collection and analysis, decision to publish, and preparation of the manuscript. JICA had no role in study design, data collection and analysis, decision to publish, or preparation of the manuscript.

There is an error in reference 1. The correct reference is: World Health Organization. Global health observatory data repository 2015: WHO; 2015 [Available from: http://www.who.int/gho/en/.]

There is an error in reference 7. The correct reference is: World Health Organization. Maternal mortality 2014; Fact sheet No. 348: WHO; 2014 [Available from: http://www.who.int/mediacentre/factsheets/fs348/en/index.html, accessed 23 November 2017.]

There is an error in reference 8. The correct reference is: United Nations Inter-agency Group for Child Mortality Estimation (UN IGME). Level and trends in child mortality. 2017.

There is an error in reference 9. The correct reference is: United Nations General Assembly. Transforming our world: the 2030 agenda for sustainable development. New York, USA: UN; 2015. Report No.: Resolution 70/1.

There is an error in reference 11. The correct reference is: World Health Organization. Home-based maternal records: Guidelines for development, adaptation and evaluation. Geneva: WHO; 1994.

There is an error in reference 12. The correct reference is: World Health Organization. Practical guide for the design, use and promotion of home-based records in immunization programmes: WHO; 2015 [Available from: http://www.who.int/immunization/monitoring_surveillance/routine/homebasedrecords/en/.]

There is an error in reference 13. The correct reference is: World Health Organization. WHO recommendations on antenatal care for a positive pregnancy experience. Geneva: WHO; 2015.

There is an error in reference 17. The correct reference is: Magwood O, Kpade V, Thavorn K, Oliver S, Mayhew AD, Pottie K. Effectiveness and cost-effectiveness of home-based records on maternal, newborn and child health outcomes: a protocol for a WHO systematic review and meta-analysis. Ottawa: Cochrane Equity Methods; 2017 [Available from: http://methods.cochrane.org/equity/projects/home-based-records.]

There is an error in reference 18. The correct reference is: World Health Organization. WHO recommendations on antenatal care for a positive pregnancy experience: WHO; 2016 [Available from: http://www.who.int/reproductivehealth/publications/maternal_perinatal_health/anc-positive-pregnancy-experience/en/.]

There is an error in reference 19. The correct reference is: World Health Organization. Infant, Newborn: WHO; 2017 [Available from: http://www.who.int/topics/infant_newborn/en/.]

There is an error in reference 21. The correct reference is: Cochrane Effective Practice and Organisation of Care (EPOC). What study designs should be included in an EPOC review? EPOC resources for review authors 2017 [Available from: epoc.cochrane.org/epoc-resources-review-authors.]

There is an error in reference 24. The correct reference is: The Cochrane Collaboration. Review Manager (RevMan) version 5.3. 5.3 ed: The Cochrane Collaboration; 2014.

There is an error in reference 35. The correct reference is: John Snow Inc. (JSI). Home-Based Record Redesigns That Worked: Lessons from Madagascar & Ethiopia. USA: JSI; 2016.

There is an error in reference 36. The correct reference is: Japan International Cooperation Agency (JICA). Study on the use of the Maternal and Child Health Handbook in the Maternal and Child Health Project–Knowledge, Lessons and Challenges. Japan: JICA; 2012.
