# Supplementary material for: Effectiveness of home-based records on maternal, newborn and child health outcomes: A systematic review and meta-analysis
Source: PLoS One. 2019 Jan 2;14(1):e0209278. doi: 10.1371/journal.pone.0209278 (PMC6314587; doi:10.1371/journal.pone.0209278)
Supplement: S1 File — (PDF) [file pone.0209278.s001.pdf]

Appendix I: Example Search Strategy

Database: Ovid MEDLINE(R) In-Process & Other Non-Indexed Citations, Ovid MEDLINE(R) Daily and Ovid MEDLINE(R) <1946 to Present>

1 exp pregnancy/
2 pregnant women/
3 exp Maternal Health Services/
4 prenatal care/
5 perinatal care/
6 postnatal care/
7 pregnan$.kw.tw
8 (antenatal or perinatal or postnatal or postpartum or prenatal or maternal or mother$) .mp.
9 exp child/
10 child$.mp.
11 exp infant/
12 infant, newborn/
13 (infanc$ or infant? or neonat$ or newborn?).mp.
14 or/1-13
15 exp Health Records, Personal/ or exp Medical Records/
16 ((health or medical or personal) adj2 record?).mp.
17 (home-based adj5 (booklet? or book? or handbook? or record? or card?)).mp.
18 (patient-held adj5 (booklet? or book? or handbook? or record? or card?)).mp.
19 (hand-held adj5 (booklet? or book? or handbook? or record? or card?)).mp.
20 (mobile adj5 (booklet? or book? or handbook? or record? or card?)).mp.
21 eHealth.kw.tw
22 mHealth.kw.tw
23 (antenatal adj5 (booklet? or book? or handbook? or record? or card?)).mp.
24 ((immunis$ or immuniz$ or vaccinat$) adj2 (booklet? or card? or record?)).mp.
25 or/15-24
26 clinical study.pt.
27 controlled clinical trial.pt.
28 clinical trial.pt.
29 randomized controlled trial.pt.
30 pragmatic clinical trial.pt.
31 random*.ab.
32 or/26-31
33 14 and 25 and 32
34 animals/ not (humans/ and animals/)
35 33 not 34
36 remove duplicates from 35
