# Supplementary material for: Effectiveness of home-based records on maternal, newborn and child health outcomes: A systematic review and meta-analysis
Source: PLoS One. 2019 Jan 2;14(1):e0209278. doi: 10.1371/journal.pone.0209278 (PMC6314587; doi:10.1371/journal.pone.0209278)
Supplement: S2 File — (PDF) [file pone.0209278.s002.pdf]

Appendix II: Table of Excluded Studies

Citation	Reason for Exclusion
Abbass-Dick J, Xie F, Koroluk J, Alcock Brillinger S, Huizinga J, Newport A, Goodman WM, Dennis CL.The Development and piloting of an eHealth breastfeeding resource targeting fathers and partners as co-parents. Midwifery. 2017 Jul;50:139-147	Study design/Intervention not relevant to PICO
Abraham S, Joshi S, Kumar V, Patwary A, Pratinidhi A, Saxena VB, Maitra K, Singh KK, Saxena NC, Saxena BN. Indian experience of home based mothers card: ICMR task force study. Indian J Pediatr. 1991 Nov-Dec;58(6):795-804.	Study design
Agrina, Arneliwati, Suyanto. The effect of reading maternal and child health's handbook campaign at posyandu (child health post) in Pekanbaru, Indonesia. The Malaysian Journal of Nursing. 2017;7(2):27-33.	Study design
Aiga H, Nguyen VD, Nguyen CD, Nguyen TTT, Nguyen THP. Knowledge, attitude and practices: assessing maternal and child health care handbook intervention in Vietnam. BMC Public Health. 2015; 16: 129.	Study design
Ainscough K, Kennelly M, Lindsay KL, O'Sullivan EJ, McAuliffe FM. Impact of an mHealth supported healthy lifestyle intervention on behavioural stage of change in overweight and obese pregnancy. 2016; Proceedings of the Nutrition Society, 75 (OCE3): E85.	Intervention not relevant to PICO
Alves C, Lasmar L, Goulart L, Alvim C, Maciel G, Viana M et al. [Quality of data on the Child Health Record and related factors]. Cad Saude Publica. 2009;25(3):583-595.	Study design
Arga M, Sahbaz H, Bakirtas A, Turktas I, Demirsoy MS.Does self-monitoring by means of symptom diaries improve asthma control in children?J Asthma. 2014 Apr;51(3):299-305	Study design/Intervention not relevant to PICO
Arnold E, Heddle N, Lane S, Sek J, Almonte T, Walker I.Handheld computers and paper diaries for documenting the use of factor concentrates used in haemophilia home therapy: a qualitative study.Haemophilia. 2005 May;11(3):216-26.	Study design/Intervention not relevant to PICO
Arora S, Peters AL, Agy C, Menchine M.A mobile health intervention for inner city patients with poorly controlled diabetes: proof-of-concept of the TExT-MED program.Diabetes Technol Ther. 2012 Jun;14(6):492-6	Intervention not relevant to PICO
Baequni. Nakamura, Y. Is maternal and child health handbook effective?: Meta-analysis of the effects of MCH Handbook. Journal of International Health. 2012, 27(2):121-127.	Study design
Balakrishnan R, Gopichandran V, Chaturvedi S, Chatterjee R, Mahapatra T, Chaudhuri I. Continuum of Care Services for Maternal and Child Health using mobile technology – a health system strengthening strategy in low and middle income countries. BMC Medical Informatics and Decision Making. 2016;16:84.	Intervention not relevant to PICO
Brown HC, Smith HJ, Mori R, Noma H.Giving women their own case notes to carry during pregnancy.Cochrane Database Syst Rev. 2015 Oct	Study design

14;(10):CD002856	
Bryant AS, Norton ME, Nakagawa S, et al. Variation in Women's Understanding of Prenatal Testing. Obstetrics and gynecology. 2015;125(6):1306-1312.	Intervention not relevant to PICO
Burbank AJ, Lewis SD, Hewes M, et al. Mobile-based asthma action plans for adolescents. The Journal of asthma : official journal of the Association for the Care of Asthma. 2015;52(6):583-586.	Study design/Intervention not relevant to PICO
Burn E, Marshall AL, Miller YD, et al The cost-effectiveness of the MobileMums intervention to increase physical activity among mothers with young children: a Markov model informed by a randomised controlled trial BMJ Open 2015;5:e007226.	Study design/Intervention not relevant to PICO
Caburnay CA, Graff K, Harris JK, et al. Evaluating Diabetes Mobile Applications for Health Literate Designs and Functionality, 2014. Preventing Chronic Disease. 2015;12:E61.	Study design/Intervention not relevant to PICO
Calderón TA, Martin H, Volpicelli K, Diaz C, Gozzer E, Buttenheim AM.Formative evaluation of a proposed mHealth program for childhood illness management in a resource-limited setting in Peru.Rev Panam Salud Publica. 2015 Aug;38(2):144-51.	Study design
Chang CW, Ma TY, Choi MS, Hsu YY, Tsai YJ, Hou TW. Electronic personal maternity records: Both web and smartphone services. Comput Methods Programs Biomed. 2015 Aug;121(1):49-58	Study design
Chen L, Du X, Zhang L, et al. Effectiveness of a smartphone app on improving immunization of children in rural Sichuan Province, China: a cluster randomized controlled trial. BMC Public Health. 2016;16(1):909.	Intervention not relevant to PICO
Choi J, Lee J hyeon, Vittinghoff E, Fukuoka Y. mHealth Physical Activity Intervention: A Randomized Pilot Study in Physically Inactive Pregnant Women. Maternal and child health journal. 2016;20(5):1091-1101.	Intervention not relevant to PICO
de Shalit N, Fattal B. The health diary as a source of information on kibbutz morbidity. Isr J Med Sci. 1990 Feb;26(2):80-7.	Intervention not relevant to PICO
Darmstadt GL, Choi Y, Arifeen SE, Bari S, Rahman SM, Mannan I, Seraji HR, Winch PJ, Saha SK, Ahmed AS, Ahmed S, Begum N, Lee AC, Black RE, Santosham M, Crook D, Baqui AH; Bangladesh Projahnmo-2 Mirzapur Study Group. Evaluation of a cluster-randomized controlled trial of a package of community-based maternal and newborn interventions in Mirzapur, Bangladesh. PLoS One. 2010 Mar 24;5(3):e9696.	Intervention not relevant to PICO
Dearlove J, Illingworth S. A controlled trial of parent initiated and conventional preschool health surveillance using personal child health records. Archives of Disease in Childhood. 1999;80(6):507-510.	Intervention not relevant to PICO
Delisle C, Sandin S, Forsum E, Henriksson H, Trolle-Lagerros Y, Larsson C, Maddison R, Ortega FB, Ruiz JR, Silfvernagel K, Timpka T, Lof M. A web- and mobile phone-based intervention to prevent obesity in 4-year-olds (MINISTOP): a population-based randomized controlled trial. BMC Public Health. 2015,15:95	Intervention not relevant to PICO

Drake AL, Unger JA, Ronen K, Matemo D, Perrier T, DeRenzi B, Richardson BA, Kinuthia J, John-Stewart G. Evaluation of mHealth strategies to optimize adherence and efficacy of Option B+ prevention of mother-to-child HIV transmission: Rationale, design and methods of a 3-armed randomized controlled trial. Contemp Clin Trials. 2017 Jun;57:44-50	Intervention not relevant to PICO
De Moraes APP, Barreto SM, Passos VMA, Golino PS, Costa JE, Vasconcelos MX. Severe maternal morbidity: a case-control study in Maranhao, Brazil. Reproductive Health. 2013;10:11.	Study design
Fedele DA, Cushing CC, Fritz A, Amaro CM, Ortega A. Mobile Health Interventions for Improving Health Outcomes in Youth: A Meta-analysis. JAMA Pediatr. 2017 May 1;171(5):461-469.	Study design
Ferrara A, Hedderson MM, Albright CL, et al. A pragmatic cluster randomized clinical trial of diabetes prevention strategies for women with gestational diabetes: design and rationale of the Gestational Diabetes' Effects on Moms (GEM) study. BMC Pregnancy and Childbirth. 2014;14:21.	Intervention not relevant to PICO
Gaskin, Gregory et al. Evaluating Parental Attitudes Towards the Acceptability and Feasibility of an Online Health Portal for Detained Youth Journal of Adolescent Health , 2015, 56(2): S58 - S59	Study design/Population not relevant to PICO
George K, Victor S, Abel R. Reliability of mother as an informant with regard to immunisation. The Indian Journal of Pediatrics. 1990, 57(4):588–590	Study design
Graham ML, Strawderman MS, Demment M, Olson CM. Does Usage of an eHealth Intervention Reduce the Risk of Excessive Gestational Weight Gain? Secondary Analysis From a Randomized Controlled Trial. Eysenbach G, ed. Journal of Medical Internet Research. 2017;19(1):e6. doi:10.2196/jmir.6644.	Intervention not related to PICO
Hagiwara A, Ueyama M, Ramlawi A, Sawada Y. Is the Maternal and Child Health (MCH) handbook effective in improving health-related behavior? Evidence from Palestine. Journal of Public Health Policy. 2013; 34(1):31-45.	Study design
Hampshire A, Blair M, Crown N, Avery A, Williams I. Action research: a useful method of promoting change in primary care? Fam Pract. 1999 Jun;16(3):305-11.	Study design
Hanna JN, Wakefield JE, Doolan CJ, Messner JL. Childhood immunisation: factors associated with failure to complete the recommended schedule by two years of age. Aust J Public Health. 1994 Mar;18(1):15-21.	Study design
Handa A, Gupta S, Tiwari VK.A comparative study of the health record cards in primary schools of Delhi. Indian Pediatr. 2008 Nov;45(11):923-5.	Intervention not relevant to PICO
Harrison D, Harker H, Heese Hd, Mann MD. An assessment by nurses and mothers of a 'Road-to-Health' Book in the Western Cape. Curationis. 2005 Nov;28(4):57-64	Study design
Hawley G, Hepworth J, Wilkinson SA, Jackson C. From maternity paper hand-held records to electronic health records: what do women tell us about their use? Aust J Prim Health. 2015 Sep 9;22(4):339-48	Study design
Hollis C, Falconer CJ, Martin JL, Whittington C, Stockton S, Glazebrook C, Davies	Study design

EB. Annual Research Review: Digital health interventions for children and young people with mental health problems – a systematic and meta-review. Journal of Child Psychology and Psychiatry. 2016; 58(4):474-503.	
Ireland AM, Wiklund I, Hsieh R, Dale P, O'Rourke E.An electronic diary is shown to be more reliable than a paper diary: results from a randomized crossover study in patients with persistent asthma.J Asthma. 2012 Nov;49(9):952-60	Intervention/population not relevant to PICO
Iskander M, Lou J, Wells M, Scarbecz M.A poster and a mobile healthcare application as information tools for dental trauma management.Dent Traumatol. 2016 Dec;32(6):457-463	Study design/Intervention not relevant to PICO
Jaddoe V, Bakker R, van Duijn C, van der Heijden A, Lindemans J, Mackenbach J et al. The Generation R Study Biobank: a resource for epidemiological studies in children and their parents. European Journal of Epidemiology. 2007;22(12):917-923.	Study design
Jan RL, Wang JY, Huang MC, Tseng SM, Su HJ, Liu LF.An internet-based interactive telemonitoring system for improving childhood asthma outcomes in Taiwan.Telemed J E Health. 2007 Jun;13(3):257-68.	Intervention not relevant to PICO
Jeffs D, Nossar V, Bailey F, Smith W, Chey T.Retention and use of personal health records: a population-based study. J Paediatr Child Health. 1994 Jun;30(3):248-52.	Study design
Jenkinson SD The entire computerized antenatal record collected prospectively and stored on optical memory cards. : Proceedings of the Silver Jubilee Congress of Obstetrics & Gynaecology. London: RCOG, 1989	Intervention not relevant to PICO
Joos O, Silva R, Amouzou A, Moulton LH, Perin J, et al. Evaluation of a mHealth Data Quality Intervention to Improve Documentation of Pregnancy Outcomes by Health Surveillance Assistants in Malawi: A Cluster Randomized Trial. PLOS ONE 2016; 11(1): e0145238	Intervention not relevant to PICO
E. Kamau-Mbuthia; S. Mbugua; Webb A. Girard; S. Kalungu; C. Sarange; W. Lou; W. Duan; C-L. Dennis; L. Nommsen-Rivers; B. Aidam; D. Sellen. Cell phone based peer counseling to support exclusive breastfeeding is associated with more frequent help and decreased breastfeeding problems. Annals of Nutrition and Metabolism. 2013; 63:196–197,	Intervention not relevant to PICO
Kaneko K, Niyonkuru J, Juma N, Mbonabuca T, Osaki K, Aoyama A. Effectiveness of the Maternal and Child Health handbook in Burundi for increasing notification of birth at health facilities and postnatal care uptake. Global Health Action. 2017;10(1):1297604.	Study design
Kawakatsu Y, Sugishita T, Oruenjo K, Wakhule S, Kibosia K, Were E, Honda S. (2015). Effectiveness of and factors related to possession of a mothers and child health handbook: an analysis using propensity score matching. Health Education Research, 1-12.	Study design
Kitabayashi H, Chiang C, Al-Shoaibi A, Hirakawa Y, Aoyama A. Association Between Maternal and Child Health Handbook and Quality of Antenatal Care Services in Palestine. Matern Child Health J. 2017;21(12):2161-2168.	Study design

Kusumayati A, Nakamura Y. Increased Utilization of Maternal Health Services by Mothers Using Maternal and Child Health Handbook in Indonesia. Journal of International Health. 2007;22(3):143-151.	Study design
M Kennelly, K Ainscough, E O'Sullivan, K Lindsay, M McCarthy, E Gibney, F McAuliffe. A randomised controlled trial of an M-health behavioural lifestyle intervention to prevent gestational diabetes in overweight and obese pregnancy: PEARS trial. BJOG-AN INTERNATIONAL JOURNAL OF OBSTETRICS AND GYNAECOLOGY. 2017; 124:14	Intervention not relevant to PICO
Kosse RC, Bouvy ML, de Vries TW, Kaptein AA, Geers HC, van Dijk L, Koster ES. mHealth intervention to support asthma self-management in adolescents: the ADAPT study. Patient Preference and Adherence, 2017; 11:571-577	Intervention not relevant to PICO
Lam J, Barr RG, Catherine N, Tsui H, Hahnhaussen CL, Pauwels J, Brant R. Electronic and paper diary recording of infant and caregiver behaviors. J Dev Behav Pediatr. 2010 Nov-Dec;31(9):685-93	Intervention not relevant to PICO
Lee SH, Nurmatov UB, Nwaru BI, Mukherjee M, Grant L, Pagliari C. Effectiveness of mHealth interventions for maternal, newborn and child health in low– and middle–income countries: Systematic review and meta–analysis. Journal of Global Health. 2016;6(1):010401.	Study design
Maher M, Hanauer DA, Kaziunas E, et al. A Novel Health Information Technology Communication System to Increase Caregiver Activation in the Context of Hospital-Based Pediatric Hematopoietic Cell Transplantation: A Pilot Study. Eysenbach G, ed. JMIR Research Protocols. 2015;4(4):e119.	Study design/Intervention not relevant to PICO
Marsh P, Kendrick D. Using a diary to record near misses and minor injuries—which method of administration is best? Injury Prevention. 1999;5(4):305-309.	Study design/Intervention not relevant to PICO
Maslowsky J, Frost S, Hendrick CE, Trujillo Cruz FO, Merajver SD. Effects of postpartum mobile phone-based education on maternal and infant health in Ecuador. Int J Gynaecol Obstet. 2016 Jul;134(1):93-8	Intervention not relevant to PICO
Mauriello LM, Van Marter DF, Umanzor CD, Castle PH, de Aguiar EL. Using mHealth to Deliver Behavior Change Interventions Within Prenatal Care at Community Health Centers. Am J Health Promot. 2016 Sep;30(7):554-62	Intervention not relevant to PICO
Mbuagbaw L, Medley N, Darzi AJ, Richardson M, Habiba Garga K, Ongolo-Zogo P. Health system and community level interventions for improving antenatal care coverage and health outcomes. The Cochrane Database of Systematic Reviews. 2015;(12):1-157.	Study design
Modi D, Gopalan R, Shah S, et al. Development and formative evaluation of an innovative mHealth intervention for improving coverage of community-based maternal, newborn and child health services in rural areas of India. Global Health Action. 2015;8:10.3402/gha.v8.26769. doi:10.3402/gha.v8.26769.	Study design
Mudany M, Sirengo M, Rutherford G, Mwangi M, Nganga L, Gichangi A. (2015). Enhancing maternal and child health using a combined mother and child health booklet in Kenya. Journal of tropical pedicatrics, 0: 1-6.	Study design
Mukanga DO; Kiguli S. Factors affecting the retention and use of child health	Study design

cards in a slum community in Kampala, Uganda, 2005. Maternal and Child Health Journal. 2006 Nov; 10(6):545-552.	
Neumayr L, Pringle S, Giles S, et al. Chart Card: Feasibility of a Tool for Improving Emergency Department Care in Sickle Cell Disease. Journal of the National Medical Association. 2010;102(11):1017-1023.	Intervention not relevant to PICO
Njoroge M, Zurovac D, Ogara EAA, Chuma J, Kirigia D. Assessing the feasibility of eHealth and mHealth: a systematic review and analysis of initiatives implemented in Kenya. BMC Research Notes. 2017;10:90. doi:10.1186/s13104-017-2416-0.	Study design
Norman GJ, Zabinski MF, Adams MA, Rosenberg DE, Yaroch AL, Atienza AA. A Review of eHealth Interventions for Physical Activity and Dietary Behavior Change. American journal of preventive medicine. 2007;33(4):336-345.	Study design
Noyes JP, Williams A, Allen D, et al. Evidence into practice: evaluating a child-centred intervention for diabetes medicine management The EPIC Project. BMC Pediatrics. 2010;10:70. doi:10.1186/1471-2431-10-70.	Study design
Oakley A, Rajan L, Robertson P. A comparison of different sources of information about pregnancy and childbirth. J Biosoc Sci. 1990 Oct;22(4):477-87.	Intervention not relevant to PICO
Oyo-Ita A, Wiysonge CS, Oringanje C, Nwachukwu CE, Oduwole O, Meremikwu MM. Interventions for improving coverage of childhood immunisation in low- and middle-income countries. The Cochrane Database of Systematic Reviews. 2016;(7):CD008145.	Study design
Pahari DP, Bastola SP, Paudel R.Factors affecting retention of child health card in a rural area.J Nepal Health Res Counc. 2011 Oct;9(2):154-8.	Study design
Palermo TM, Valenzuela D, Stork PP. A randomized trial of electronic versus paper pain diaries in children: impact on compliance, accuracy, and acceptability. Pain. 2004 Feb;107(3):213-9.	Intervention not relevant to PICO
Patel SJ, Longhurst CA, Lin A, Garrett L, Gillette-Arroyo J, Mark JD, Wood MS, Sharek PJ. Integrating the home management plan of care for children with asthma into an electronic medical record.Jt Comm J Qual Patient Saf. 2012 Aug;38(8):359-65.	Study design/Intervention not relevant to PICO
Pawellek I, Richardsen T, Oberle D, Grote V, Koletzko B. Use of electronic data capture in a clinical trial on infant feeding. Eur J Clin Nutr. 2012 Dec;66(12):1342-3.	Intervention not relevant to PICO
Phipps H. Carrying their own medical records: the perspective of pregnant women. Aust N Z J Obstet Gynaecol. 2001 Nov;41(4):398-401.	Study design
Radhakrishna K, Goud BR, Kasthuri A, Waghmare A, Raj T. Electronic Health Records and Information Portability: A Pilot Study in a Rural Primary Healthcare Center in India. Perspectives in Health Information Management. 2014;11(Summer):1b.	Study design/Intervention not relevant to PICO
Ralston JD, Carrell D, Reid R, Anderson M, Moran M, Hereford J. Patient Web Services Integrated with a Shared Medical Record: Patient Use and Satisfaction.	Study design

Journal of the American Medical Informatics Association : JAMIA. 2007;14(6):798-806.	
Roberfroid D, Pelto GH, Kolsteren P. Plot and see! Maternal comprehension of growth charts worldwide. Tropical Medicine and International Health. 2007, 12(9):1074-1086.	Study design
Rossdale M, Clark C, James J. Improved health care delivery in an inner-city well-baby clinic run by general practitioners. The Journal of the Royal College of General Practitioners. 1986;36(292):512-513.	Intervention not relevant to PICO
Rowe RE, Garcia J, Macfarlane AJ, Davidson LL. Improving communication between health professionals and women in maternity care: a structured review. Health Expectations : An International Journal of Public Participation in Health Care and Health Policy. 2002;5(1):63-83.	Study design
Teng Liaw, Martin Lawrence, Jenny Rendell; The effect of a computer-generated patient-held medical record summary and/or a written personal health record on patients' attitudes, knowledge and behaviour concerning health promotion. Fam Pract 1996; 13 (3): 289-293.	Population not relevant to PICO
Shah PM, Selwyn BJ, Shah K, Kumar V. Evaluation of the home-based maternal record: a WHO collaborative study. Bulletin of the World Health Organization. 1993;71(5):535-548.	Study design
Shiferaw S, Spigt M, Tekie M, Abdullah M, Fantahun M, Dinant G-J. The Effects of a Locally Developed mHealth Intervention on Delivery and Postnatal Care Utilization; A Prospective Controlled Evaluation among Health Centres in Ethiopia. Gebhardt G, ed. PLoS ONE. 2016;11(7):e0158600.	Study design
Simba DO. Towards a sustainable community database: taking advantage of the Road-to-Health cards to monitor and evaluate health interventions targeting under fives. Tanzan J Health Res. 2009 Jan;11(1):46-50.	Study design
Sistiarani C, Dardjito E, Nurhayati S. Educational Leaflet to improve mothers knowledge about utilization of "maternal and child health book" in Kalibagor, Indonesia. Management in Health. 2015;19(1).	Study design
Sitaresmi MN, Mostert S, Gundy CM, Ismail D, Veerman AJ. A medication diary-book for pediatric patients with acute lymphoblastic leukemia in Indonesia. Pediatr Blood Cancer. 2013 Oct;60(10):1593-7.	Intervention not relevant to PICO
Sondaal SFV, Browne JL, Amoakoh-Coleman M, et al. Assessing the Effect of mHealth Interventions in Improving Maternal and Neonatal Care in Low- and Middle-Income Countries: A Systematic Review. Li D, ed. PLoS ONE. 2016;11(5):e0154664. doi:10.1371/journal.pone.0154664.	Study design
Stevens MM. "Shuttle sheet": a patient-held medical record for pediatric oncology families. Med Pediatr Oncol. 1992;20(4):330-	Study design
Theron GB. Effect of the maternal care manual of the perinatal education programme on the ability of midwives to interpret antenatal cards and partograms. J Perinatol. 1999 Sep;19(6 Pt 1):432-5.	Population not relevant to PICO
Thompson SC, Kennedy RI.Documentation of children's vaccination status in	Study design

child care centres in Victoria. J Paediatr Child Health. 1998 Apr;34(2):175-8.	
Thorsdottir I, Torfadottir JE, Birgisdottir BE, Geirsson RT. Weight gain in women of normal weight before pregnancy: complications in pregnancy or delivery and birth outcome. Obstet Gynecol. 2002 May;99(5 Pt 1):799-806	Study design/Intervention not relevant to PICO
Turner KE, Fuller S. Patient-Held Maternal and/or Child Health Records: Meeting the Information Needs of Patients and Healthcare Providers in Developing Countries? Online J Public Health Inform. 2011;3(2).	Study design
Van Dijk MR, Huijgen NA, Willemsen SP, Laven JS, Steegers EA, Steegers-Theunissen RP. Impact of an mHealth Platform for Pregnancy on Nutrition and Lifestyle of the Reproductive Population: A Survey. Morita P, ed. JMIR mHealth and uHealth. 2016;4(2):e53. doi:10.2196/mhealth.5197.	Study design/Intervention not relevant to PICO
Vincelet C, Tabone MD, Berthier M, Bonnefoi MC, Chevallier B, Lemaire JP, Dommergues JP. How are personal child health records completed? A multicentric evaluation study. Archives de Pediatrie : Organe Officiel de la Societe Francaise de Pediatrie. 2003, 10(5):403-409	Study design
Zhou YQ, Fan CX, Zheng JS. Study on comprehensive strategies of strenghening routine immunization program. Zhongguo Yi Miao He Mian Yi. 2009 Oct;15(5):459-61	Full text could not be retrieved
