# Supplementary material for: Effectiveness of home-based records on maternal, newborn and child health outcomes: A systematic review and meta-analysis
Source: PLoS One. 2019 Jan 2;14(1):e0209278. doi: 10.1371/journal.pone.0209278 (PMC6314587; doi:10.1371/journal.pone.0209278)
Supplement: S3 File — (PDF) [file pone.0209278.s003.pdf]

Appendix III: GRADE Evidence Profiles

1. For women during pregnancy and after birth, and for newborns, children and caregivers (P), does use of any home-based records (I), compared with no use of any home-based records (C), improve maternal, newborn and child health outcomes (O)?

1.1 Maternal health

a. Maternal care seeking

Quality assessment							No. of participants		Effect		Certainty (GRADE)	Importance
No. of studies	Study design	Risk of bias	Inconsistency	Indirectness	Imprecision	Other considerations	# Events/ Intervention	# Events/ Control	Relative (95% CI)	Absolute (95% CI)		
Antenatal care visits: average number of visits<br>Studies: Mori, 2015 (Mongolia); Osaki, 2018 (Indonesia)												
2	cRCTs	Serious[1]	Not serious	Serious[2]	Not serious	None	Mori (2015): Mean 6.615 (± 1.525)<br>Osaki (2018): Mean 6.3 (± 2.5)	Mori (2015): Mean 6.407 (± 1.765)<br>Osaki (2018): Mean 5.6 (± 3.1)	Mori (2015): mean difference 0.208 (-0.710 to 1.125)	Not calculated	LOW	Critical
Antenatal care visits: six or more visits<br>Studies: Mori, 2015 (Mongolia); Osaki, 2018 (Indonesia)												
2	cRCTs	Serious[1]	Serious[3]	Serious[2]	None	None	306/436	285/519	OR 1.93 (1.48 to 2.53)	152 more per 1000 (from 94 more to 206 more)	VERY LOW	Critical
Antenatal care visits: four or more visits<br>Studies: Osaki, 2018 (Indonesia)												
1	cRCT	Serious[1]	Not serious	Not serious	Not serious	None	133/183	185/271	OR 1.25 (0.81 to 1.95)	Not calculated	MODERATE	Critical
Care seeking for pregnancy complications<br>Studies: Osaki, 2018 (Indonesia)												
1	cRCT	Serious[1]	Not serious	Not serious	Very serious[4]	None	11/13	36/53	OR 2.6 (0.52 to 13.04)	Not calculated	VERY LOW	Critical

Maternal immunization: TT2<br>Studies: Osaki, 2018 (Indonesia)												
1	cRCT	Serious[1]	Not serious	Not serious	Not serious	None	139/183	162/271	OR 1.98<br>(1.29 to 3.04)	Not calculated	MODERATE	Critical
Childbirth with a skilled birth attendant at a health facility<br>Studies: Osaki, 2018 (Indonesia)												
1	cRCT	Serious[1]	Not serious	Not serious	Serious[5]	None	79/183	106/271	OR 1.14<br>(0.75 to 1.74)	Not calculated	LOW	Critical
Care seeking for postpartum complications<br>Studies: Osaki, 2018 (Indonesia)												
1	cRCT	Serious[1]	Not serious	Not serious	Very serious[6]	None	4/6	8/28	OR 5.0<br>(0.76 to 32.93)	Not calculated	VERY LOW	Critical

[1] Allocation concealment and attrition bias
[2] Differences in comparison groups (sporadic availability of HBRs vs delay of 7 months)
[3] Mori (2015) reports no effect on outcome; Osaki (2018) reports significant effect
[4] Very low number of events (<100) and wide confidence intervals
[5] Low number of events (<300)
[6] Low number of events (<300) and wide confidence intervals

b. Maternal self-care practices

Quality assessment							No. of participants		Effect		Certainty (GRADE)	Importance
No. of studies	Study design	Risk of bias	Inconsistency	Indirectness	Imprecision	Other considerations	# Events/ Intervention	# Events/ Control	Relative	Absolute		
Healthy pregnancy behaviours: smoking during pregnancy<br>Studies: Mori, 2015 (Mongolia)												
1	cRCT	Serious[1]	Not serious	Not serious	Very serious[2]	12 control participants received the intervention	5/253	7/247	RR 1.01[3]<br>(0.9 to 1.04)	Not calculated	VERY LOW	Critical
Healthy pregnancy behaviours: drinking during pregnancy<br>Studies: Mori, 2015 (Mongolia)												
1	cRCT	Serious[1]	Not serious	Not serious	Very serious[2]	12 control participants	20/251	35/248	RR 1.07[4]<br>(0.97 to 1.18)	Not calculated	VERY LOW	Critical

						received the intervention						

Healthy household environment: smoking among family members<br>Studies: Mori, 2015 (Mongolia)												
1	cRCT	Serious[1]	Not serious	Not serious	Serious[5]	12 control participants received the intervention	129/252	151/247	RR 0.84[4] (0.7 to 0.99)	97 fewer per 1000 (from 6 to 177 fewer)	LOW	Critical
Improved communication within the household: husband's support (proxy)<br>Studies: Osaki, 2018 (Indonesia)												
1	cRCT	Serious[6]	Not serious	Serious[7]	Serious[5]	None	109/183	119/271	OR 1.82 (1.20 to 2.76)	157 more per 1000 (from 64 to 249 more)	LOW	Critical

[1] Serious concerns regarding confounding
[2] Very low number of events (<100)
[3] In Mori (2015) 12 control participants received the intervention
[4] Risk Ratio
[5] Low number of events (<300)
[6] Allocation concealment and attrition bias in Osaki (2018)
[7] Proxy outcome (indirect evidence)

c. Maternal mortality and morbidity

Quality assessment							No. of participants		Effect		Certainty (GRADE)	Importance
No. of studies	Study design	Risk of bias	Inconsistency	Indirectness	Imprecision	Other considerations	Intervention	Control	Relative	Absolute		
Postnatal depression<br>Studies: Mori, 2015 (Mongolia)												
1	cRCT	Serious[1]	Not serious	Not serious	Very serious[2]	12 control participants received the intervention	15/253	11/248	RR 0.99[3] (0.94 to 1.04)	Not calculated	VERY LOW	Important

[1] Serious concerns regarding confounding
[2] Very low number of events (<100)
[3] Risk Ratio

1.2 Newborn health

a. Newborn care seeking

Quality assessment							No. of participants		Effect		Certainty (GRADE)	Importance
No. of studies	Study design	Risk of bias	Inconsistency	Indirectness	Imprecision	Other considerations	Intervention	Control	Relative	Absolute		
Care seeking for newborn illness<br>Studies: Osaki, 2018 (Indonesia)												
1	cRCT	Serious[1]	Not serious	Not serious	Very serious[2]	None	10/14	17/29	OR 1.76<br>(0.45 to 6.98)	Not calculated	VERY LOW	Critical

[1] Allocation concealment and attrition bias in Osaki (2018)
[2] Very low number of events (<100)

b. Newborn care practices

Quality assessment							No. of participants		Effect		Certainty (GRADE)	Importance
No. of studies	Study design	Risk of bias	Inconsistency	Indirectness	Imprecision	Other considerations	Intervention	Control	Relative	Absolute		
Immediate breastfeeding<br>Studies: Mori, 2015 (Mongolia)												
1	cRCT	Serious[1]	Not serious	Not serious	Not serious	12 control participants received the intervention	252/253	244/246	RR 1.07[2]<br>(0.97 to 1.18)	Not calculated	MODERATE	Critical
Improved communication within the household: husband's support (proxy)<br>Studies: Osaki, 2018 (Indonesia)												
1	cRCT	Serious[3]	Not serious	Serious[4]	Serious[5]	None	65/183	72/271	OR 1.58<br>(1.02 to 2.46)	89 more per 1000 (from 3 to 176 more)	VERY LOW	Important

[1] Serious concerns regarding confounding
[2] Risk Ratio
[3] Allocation concealment and attrition bias in Osaki (2018)
[4] Proxy outcome (indirect evidence)
[5] Low number of events (<300)

c. Perinatal mortality and morbidity

Quality assessment							No. of participants		Effect		Certainty (GRADE)	Importance
No. of studies	Study design	Risk of bias	Inconsistency	Indirectness	Imprecision	Other considerations	Intervention	Control	Relative	Absolute		
Neonatal deaths<br>Studies: Mori, 2015 (Mongolia)												
1	cRCT	Serious[1]	Not serious	Not serious	Very serious[2]	12 control participants received the intervention	1/253	2/248	RR 1.00[3]<br>(0.99 to 1.02)	Not calculated	VERY LOW	Important
APGAR score<br>Studies: Mori, 2015 (Mongolia)												
1	cRCT	Serious[1]	Not serious	Not serious	Not serious	12 control participants received the intervention	Mean: 7.55<br>(± 0.89)	Mean: 7.34<br>(± 1.25)	Mean difference: 0.210<br>(0.212 to 0.632)	Not calculated	MODERATE	Important

[1] Serious concerns regarding confounding
[2] Very low number of events (<100)
[3] Risk Ratio

1.3 Child health

a. Vaccination use

Quality assessment							No. of participants		Effect		Certainty (GRADE)	Importance
No. of studies	Study design	Risk of bias	Inconsistency	Indirectness	Imprecision	Other considerations	Intervention	Control	Relative (95% CI)	Absolute (95% CI)		
DTP3 completion<br>Studies: Lakhani, 1984 (UK); Stille, 2001 (US)												
2	RCT (1)<br>Non-randomized controlled trial (1)	Very serious[1]	Not serious	Serious[2]	Not serious	None	126/313	136/301	OR 0.82<br>(0.52 to 1.30)	Not calculated	VERY LOW	Critical

[1] Stille (2001) non-randomized design and selection bias

[2] Differences in DTP completion measurement and differences in intervention design

b. Child care seeking

Quality assessment							No. of participants		Effect		Certainty (GRADE)	Importance
No. of studies	Study design	Risk of bias	Inconsistency	Indirectness	Imprecision	Other considerations	Intervention	Control	Relative (95% CI)	Absolute (95% CI)		
Care seeking for childhood illness<br>Studies: Osaki, 2018 (Indonesia)												
1	cRCT	Serious[1]	Not serious	Not serious	Very serious[2]	None	Not reported	Not reported	Not reported	"Care seeking from health personnel was similarly observed in both areas"	VERY LOW	Critical
Care seeking for childhood illness: frequency of contact with health services<br>Studies: Bjerkeli Grovdal, 2006 (Norway)												
1	RCT	Serious[3]	Not serious	Not serious	Very serious[4]	None	Children with more encounters with healthcare services<br>Non-routine child health centre: 35/155<br>Doctor outside child health centre: 30/155<br>Specialist or hospital: 13/155	Children with more encounters with healthcare services<br>Non-routine child health centre: 35/154<br>Doctor outside child health centre:28/154<br>Specialist or hospital: 16/154	Non-routine child health centre:<br>OR 0.99 (0.58 to 1.69)<br>Doctor outside child health centre:<br>OR 1.08 (0.61 to 1.91)<br>Specialist or hospital:<br>OR 1.25 (0.37 to 1.7)	Not calculated	VERY LOW	Critical
Care seeking for childhood illness: children with chronic disease<br>Studies: Bjerkeli Grovdal, 2006 (Norway)												
1	RCT	Serious[3]	Not serious	Serious[5]	Very serious[6]	None	Not reported	Not reported	Not reported	"17% more parents in	VERY LOW	Critical

										the control group visited the child health centre”		

[1] Allocation concealment and attrition bias
[2] Data not reported
[3] High risk for section bias
[4] Unable to assess number of events as outcome data are ordinal
[5] Population is children with chronic illness for this outcome
[6] Unable to assess number of events as not reported

c. Child care practices

Quality assessment							No. of participants		Effect		Certainty (GRADE)	Importance
No. of studies	Study design	Risk of bias	Inconsistency	Indirectness	Imprecision	Other considerations	Intervention	Control	Relative	Absolute		
Exclusive breastfeeding<br>Studies: Osaki, 2018 (Indonesia)												
1	cRCT	Serious[1]	Not serious	Not serious	Serious[2]	None	79/183	132/271	OR 0.76 (0.51 to 1.14)	Not calculated	LOW	Critical
Complementary feeding<br>Studies: Osaki, 2018 (Indonesia)												
1	cRCT	Serious[1]	Not serious	Not serious	Serious[2]	Large effect noted in a positive direction	113/183	74/271	OR 4.35 (2.85 to 6.65)	344 more per 1000 (from 256 to 433 more)	MODERATE	Critical
Continued breastfeeding<br>Studies: Osaki, 2018 (Indonesia)												
1	cRCT	Serious[1]	Not serious	Not serious	Not serious	None	167/183	224/271	OR 2.31 (1.22 to 4.39)	86 more per 1000 (from 25 to 146 more)	MODERATE	Critical
Infant and child illness management: Vitamin A use<br>Studies: Osaki, 2018 (Indonesia)												
1	cRCT	Serious[1]	Not serious	Not serious	Not serious	None	160/183	205/271	OR 2.00	118 more per 1000 (from 47	MODERATE	Critical

									(1.16 to 3.47)	to 188 more)		
Infant and child illness management: home care cough<br>Studies: Osaki, 2018 (Indonesia)												
1	cRCT	Serious[1]	Not serious	Not serious	Very serious[3]	Large effect noted in a positive direction	36/45	32/60	OR 3.50 (1.44 to 8.52)	267 more per 1000 (from 89 more to 374 more)	LOW	Critical
Infant and child illness management: home care diarrhoea<br>Studies: Osaki, 2018 (Indonesia)												
1	cRCT	Serious[1]	Not serious	Serious[4]	Very serious[3]	None	20/24	25/27	Not reported	Not calculated	VERY LOW	Critical
Improved communication within the household: husband's support (proxy)<br>Studies: Osaki, 2015 (Indonesia)												
1	cRCT	Serious[1]	Not serious	Serious[5]	Serious[2]	None	78/183	86/271	OR 1.62 (1.06 to 2.48)	109 more per 1000 (from 18 to 200 more)	VERY LOW	Important

[1] Allocation concealment and attrition bias in Osaki (2018)
[2] Low number of events (<300)
[3] Very low number of events (<100)
[4] Diarrhoea only one of many possibly illnesses
[5] Proxy outcome (indirect evidence)

d. Child mortality and morbidity

Quality assessment							No. of participants		Effect		Certainty (GRADE)	Importance
No. of studies	Study design	Risk of bias	Inconsistency	Indirectness	Imprecision	Other considerations	Intervention	Control	Relative	Absolute		
Underweight children<br>Studies: Osaki, 2018 (Indonesia)												
1	cRCT	Serious[1]	Not serious	Not serious	Very serious[2]	None	7/135	35/250	OR 0.33<br>(0.12 to 0.94)	88 fewer per 1000 (from 31 to 145 fewer)	VERY LOW	Important
Stunted growth<br>Studies: Osaki, 2018 (Indonesia)												
1	cRCT	Serious[1]	Not serious	Not serious	Serious[3]	None	35/133	100/248	OR 0.53<br>(0.30 to 0.92)	140 fewer per 1000 (from 44 to 237 fewer)	LOW	Important
Wasting<br>Studies: Osaki, 2018 (Indonesia)												
1	cRCT	Serious[1]	Not serious	Not serious	Very serious[2]	None	10/133	30/248	OR 0.59<br>(0.24 to 1.47)	Not calculated	VERY LOW	Important
Risk of cognitive delay<br>Studies: Dagvadorj, 2017 (Mongolia)												
1	cRCT	Very serious[4]	Not serious	Not serious	Very serious[2]	None	17/214	24/172	OR 0.32<br>(0.14 to 0.73)	90 fewer per 1000 (from 34 to 117 fewer)	VERY LOW	Important

[1] Allocation concealment and attrition bias
[2] Very low number of events (<100)
[3] Low number of events (<300)
[4] High risk for performance, detection and attrition bias; participants were not blinded to intervention

1.4 Care seeking across the MNCH continuum

Quality assessment							No. of participants		Effect		Certainty (GRADE)	Importance
No. of studies	Study design	Risk of bias	Inconsistency	Indirectness	Imprecision	Other considerations	Intervention	Control	Relative	Absolute		
Maternal: TT2, ANC4, SBA<br>Studies: Osaki, 2018 (Indonesia)												
1	cRCT	Serious[1]	Not serious	Not serious	Serious[2]	None	53/183	50/271	OR 1.46 (0.89 to 2.40)	Not calculated	LOW	Important
Maternal and newborn: TT2, ANC4, SBA, VitA, ExBF<br>Studies: Osaki, 2018 (Indonesia)												
1	cRCT	Serious[1]	Not serious	Not serious	Very serious[3]	None	31/183	22/271	OR 2.38 (1.22 to 4.64)	88 more per 1000 (from 24 to 151 more)	VERY LOW	Important
Maternal, newborn and child: TT2, ANC4, SBA, VitA, ExBF, started CF in 6-9 months<br>Studies: Osaki, 2018 (Indonesia)												
1	cRCT	Serious[1]	Not serious	Not serious	Very serious[3]	Large effect noted in a positive direction, however wide CIs	22/183	5/271	OR 7.13 (2.43 to 20.90)	100 more per 1000 (from 25 to 264 more)	LOW	Important

[1] Allocation concealment and attrition bias
[2] Low number of events (<300)
[3] Very low number of events (<100) and wide confidence intervals

2. For women during pregnancy and after birth, and for newborns, children and caregivers (P), does use of any home-based records (I), compared with inconsistent use (low use) of any home-based records (C), improve maternal, newborn and child health outcomes (O)?

(No studies)

3. For women during pregnancy and after birth, and for newborns, children and caregivers (P), does use of different types of home-based records (I) and (C), improve maternal, newborn and child health outcomes (O)?

3.1 Maternal health

a. Maternal care seeking

Quality assessment							No. of participants		Effect		Certainty (GRADE)	Importance
No. of studies	Study design	Risk of bias	Inconsistency	Indirectness	Imprecision	Other considerations	Intervention	Control	Relative (95% CI)	Absolute (95% CI)		
Antenatal care visits: % of women attending four or more ANC visits<br>Studies: Yanagisawa, 2015 (Cambodia)												
1	Non-randomized controlled trial	Serious[1]	Not serious	Not serious	Not serious	None	Pre-intervention survey: 33.1%<br>Post-intervention survey: 45.3%<br>Difference: 12.3%	Pre-intervention survey: 29.4%<br>Post-intervention survey: 39.7%<br>Difference: 10.3%	Difference-in-Differences: 1.9%<br>Adjusted OR (intervention): 1.55<br>(1.09 to 2.20)<br>Adjusted OR (control): 1.28<br>(0.90 to 1.81)	Not calculated	VERY LOW	Critical
Missed antenatal care appointments:<br>Studies: Lovell, 1987 (UK)												
1	RCT	Serious[2]	Not serious	Not serious	Serious[3]	"Mothers in the control group also had access to their notes while waiting in antenatal clinic"	73/98	65/105	OR 1.8<br>(0.99 to 3.28)	Not calculated	LOW	Critical

Childbirth with a skilled birth attendant<br>Studies: Yanagisawa, 2015 (Cambodia)												
1	Non-randomized controlled trial	Serious[1]	Not serious	Not serious	Not serious	None	Pre-intervention survey: 53.8%<br>Post-intervention survey: 77.2%<br>Difference: 23.4%	Pre-intervention survey:56.6%<br>Post-intervention survey: 67.8%<br>Difference: 11.2%	Difference-in-differences: 12.2%<br>Adjusted OR (intervention): 2.613<br>(1.81 to 3.78)<br>Adjusted OR (control): 1.09<br>(0.76 to 1.56)	Not calculated	VERY LOW	Critical

[1] High risk for selection, performance and detection bias
[2] High risk for selection, performance, detection and attrition bias
[3] Less than 300 events

b. Maternal care practices

Quality assessment							No. of participants		Effect		Certainty (GRADE)	Importance
No. of studies	Study design	Risk of bias	Inconsistency	Indirectness	Imprecision	Other considerations	Intervention	Control	Relative (95% CI)	Absolute (95% CI)		
Healthy pregnancy behaviours: smoking at 8-16 and 32-34 weeks<br>Studies: Lovell, 1987 (UK)												
1	RCT	Serious[1]	Not serious	Not serious	Serious[2]	"Mothers in the control group also had access to their notes while waiting in antenatal clinic"	8-16 wks: 74/98<br>32-34 wks: 73/98	8-16 wks: 79/105<br>32-34 wks: 77/105	8-16 wks:<br>OR 1.01<br>(0.54 to 1.92)<br>32-34 wks: OR 1.06<br>(0.57 to 1.99)	Not calculated	LOW	Critical
Healthy pregnancy behaviours: number of cigarettes smoked<br>Studies: Elbourne,1987 (UK)												
1	RCT	Serious[1]	Not serious	Not serious	Very serious[3]	None	Not reported	Not reported	Not reported	"Clinical outcomes and women's health-related behaviour did not exhibit statistically	VERY LOW	Critical

										significant differences either between the two groups overall, or in terms of 'within-person' changes over the time period in the number of cigarettes smoked."		
Healthy pregnancy behaviours: drinking at 8-16 and 32-34 weeks<br>Studies: Lovell,1987 (UK)												
1	RCT	Serious[1]	Not serious	Not serious	Serious[4]	Mothers in the control group also had access to their notes while waiting in antenatal clinic	8-16 wks: 65/98<br>32-34 wks: 59/98	8-16 wks: 77/105<br>32-34 wks: 72/105	OR 0.72 (0.39 to 1.31)<br>OR 0.69 (0.39 to 1.24)	Not calculated	LOW	Critical

[1] High risk for selection, concealment, detection and attrition bias
[2] Less than 300 events
[3] No data reported
[4] Less than 300 events

c. Maternal mortality and morbidity

Quality assessment							No. of participants		Effect		Certainty (GRADE)	Importance
No. of studies	Study design	Risk of bias	Inconsistency	Indirectness	Imprecision	Other considerations	Intervention	Control	Relative (95% CI)	Absolute (95% CI)		
Clinical outcomes of the mother<br>Studies: Lovell, 1987 (UK)												
1	RCT	Serious[1]	Not serious	Serious[2]	Serious[3]	None	55/104	69/108	OR 0.63 (0.37 to 1.1)	Not calculated	VERY LOW	Important

[1] High risk for selection, performance, detection and attrition bias
[2] Population comprised of a higher proportion of one-parent families, high unemployment rate and a quarter of sample included West Indian and other groups disproportionately affected by social-deprivation
[3] Small sample size (less than 300 events)

3.2 Newborn health

a. Newborn care practices

Quality assessment							No. of participants		Effect		Certainty (GRADE)	Importance
No. of studies	Study design	Risk of bias	Inconsistency	Indirectness	Imprecision	Other considerations	Intervention	Control	Relative	Absolute		
Early breastfeeding: Percentage of participants that initiated early breastfeeding<br>Studies: Yanagisawa, 2015 (Cambodia)												
1	Non-randomized controlled trial	Serious[1]	Not serious	Not serious	Serious[2]	None	Pre-intervention survey: 23.8%<br>Post intervention survey: 40.0%<br>Difference:16.2%	Pre-intervention survey: 30.0%<br>Post-intervention survey: 40.0%<br>Difference: 10%	Difference-in-differences: 6.2%<br>OR not reported	Not calculated	VERY LOW	Critical
Immediate breastfeeding<br>Studies: Lovell, 1987 (UK)												
1	Non-randomized controlled trial	Serious[1]	Not serious	Serious[3]	Serious[4]	None	77/98	81/105	OR 1.09 (0.56 to 2.11)	Not calculated	VERY LOW	Critical

[1] High risk for selection, performance, detection and attrition bias
[2] Sample size and event numbers not available
[3] Population comprised of a higher proportion of one-parent families, high unemployment rate and a quarter of sample included West Indian and other groups disproportionately affected by social-deprivation
[4] Small sample sizes (less than 300 events)

b. Improved communication within the household

Quality assessment							No. of participants		Effect		Certainty (GRADE)	Importance
No. of studies	Study design	Risk of bias	Inconsistency	Indirectness	Imprecision	Other considerations	Intervention	Control	Relative	Absolute		
Improved communication within the household: husband support (proxy)<br>Studies: Elbourne, 1987 (UK)												
1	RCT	Serious[1]	Not serious	Serious[2]	Very serious[3]	None	Not reported	Not reported	Not reported	Not calculated	VERY LOW	Important

[1] High risk for selection, performance, detection and attrition bias

[2] Proxy outcome (indirect evidence)
[3] Unable to assess as number of events not reported

c. Perinatal mortality and morbidity

Quality assessment							No. of participants		Effect		Certainty (GRADE)	Importance
No. of studies	Study design	Risk of bias	Inconsistency	Indirectness	Imprecision	Other considerations	Intervention	Control	Relative	Absolute		
Neonatal deaths or stillbirths<br>Studies: Lovell, 1987 (UK)												
1	RCT	Serious[1]	Not serious	Serious[2]	Very serious[3]	None	2/104	2/108	OR 1.04 (0.1 to 7.52)	Not calculated	VERY LOW	Important
Newborn outcomes (complications in the baby and stillborn or newborn death)<br>Studies: Lovell, 1987 (UK)												
1	RCT	Serious[1]	Not serious	Serious[2]	Very serious[4]	None	Major antenatal complication, complications with the baby, miscarriage, stillborn or neonatal death: 49/104	Major antenatal complication, complications with the baby, miscarriage, stillborn or neonatal death: 39/108	OR 1.58 (0.91 to 2.73)	Not calculated	VERY LOW	Important

[1] High risk for selection, performance, detection and attrition bias
[2] Population comprised of a higher proportion of one-parent families, high unemployment rate and a quarter of sample included West Indian and other groups disproportionately affected by social-deprivation
[3] Wide confidence interval and small number of events
[4] Less than 100 events

3.3 Child health

a. Vaccination uptake

Quality assessment							No. of participants		Effect		Certainty (GRADE)	Importance
No. of studies	Study design	Risk of bias	Inconsistency	Indirectness	Imprecision	Other considerations	Intervention	Control	Relative	Absolute		
DTP3 completion<br>Studies: Usman,2009 (Pakistan); Usman, 2011 (Pakistan)												

2	RCTs	Not serious	Serious[1]	Not serious	Not serious	None	511/753	354/753	OR 2.39 (1.45 to 3.92)	209 more per 1000 (from 93 to 307 more)	MODERATE	CRITICAL

[1] I^2 value of 82% suggests high heterogeneity between studies

4. For women during pregnancy and after birth, and for caregivers (P), does any use of home-based records (I), compared with no use of any home-based records (C), improve health service outcomes (O)?

4.1 Quality of care

a. Communication between women/caregivers and health providers

Quality assessment							No. of participants		Effect		Certainty (GRADE)	Importance
No. of studies	Study design	Risk of bias	Inconsistency	Indirectness	Imprecision	Other considerations	Intervention	Control	Relative (95% CI)	Absolute (95% CI)		
Communication: difficulty talking to health personnel (proxy)<br>Studies: Bjerkeli Grovdal, 2006 (Norway)												
1	RCT	Serious[1]	Not serious	Serious[2]	Very serious[3]	None	Parents with more difficulty talking to health personnel:<br>Nurse 8/119<br>Doctor 19/118<br>Other doctors 16/89<br>Other health personnel 1/24	Parents with more difficulty talking to health personnel:<br>Nurse 11/115<br>Doctor 17/122<br>Other doctors 12/104<br>Other 6/47	Ordinal outcome measure:<br>Nurse p=0.86<br>Doctor p=0.78<br>Other doctors p=0.39<br>Other p=0.60	Not calculated	VERY LOW	Important
Communication: influence on communication (proxy)<br>Studies: Moore, 2000 (UK)												
1	RCT	Very serious[4]	Not serious	Serious[2,5]	Very serious[6]	None	Not reported	Not reported	Not reported	"With one exception. there was no indication of a change [in communication] after	VERY LOW	Important

										using the record”		
Communication: received explanation from health personnel (proxy)<br>Studies: Osaki, 2018 (Indonesia)												
1	cRCT	Serious[6]	Not serious	Serious[2]	Serious[7]	Serious concern[8]	Improvement from baseline: 131/183	Improvement from baseline: 31/271	Difference in differences: 60.1%	There was a 60.1% higher increase in the people who had ever received explanation in the intervention arm compared to the control. No statistics reported comparing the two groups	VERY LOW	Important

[1] High risk for section bias
[2] Proxy outcome (indirect evidence)
[3] Unable to assess number of events as outcome data are ordinal
[4] High risk for selection, attrition and other bias
[5] Population is children with disabilities
[6] Number of cases not reported
[6] Allocation concealment and attrition bias in Osaki (2018)
[7] Low number of events (<300)
[8] Comparison group had higher values at baseline

b. Satisfaction with services

Quality assessment							No. of participants		Effect		Certainty (GRADE)	Importance
No. of studies	Study design	Risk of bias	Inconsistency	Indirectness	Imprecision	Other considerations	Intervention	Control	Relative (95% CI)	Absolute (95% CI)		
Satisfaction with information provided (proxy)<br>Studies: Bjerkeli Grovdal, 2006 (Norway)												
1	RCT	Serious[1]	Not serious	Serious[2]	Very serious[3]	None	Not reported	Not reported	Not reported	"Parental satisfaction with information provided about their child's health from different professionals was the same in both groups"	VERY LOW	Important

[1] High risk for section bias
[2] Proxy outcome (indirect evidence)
[3] Number of cases not reported

c. Continuity of care

Quality assessment							No. of participants		Effect		Certainty (GRADE)	Importance
No. of studies	Study design	Risk of bias	Inconsistency	Indirectness	Imprecision	Other considerations	Intervention	Control	Relative (95% CI)	Absolute (95% CI)		
Continuity of care after a two-year follow-up: brought to more than two facilities<br>Studies: Osaki, 2018 (Indonesia)												
1	cRCT	Serious[1]	Not serious	Not serious	Serious[2]	Serious concern[3]	Improvement from baseline: 94/183	Improvement from baseline: 17/271	Difference in differences 45%	Not calculated	VERY LOW	Important
Continuity of care after a two-year follow-up: brought to more than two occasions<br>Studies: Osaki, 2018 (Indonesia)												
1	cRCT	Serious[1]	Not serious	Not serious	Serious[2]	Serious	Improvement	Improvement	Difference in	Not	VERY LOW	Important

						concern[3]	from baseline: 95/183	from baseline: 36/271	differences 38.6%	calculated		
Continuity of care after a two-year follow-up: filled in by more than two personnel<br>Studies: Osaki, 2018 (Indonesia)												
1	cRCT	Serious[1]	Not serious	Not serious	Serious[2]	Serious concern[3]	Improvement from baseline: 76/183	Improvement from baseline: 24/271	Difference in differences 33.7%	Not calculated	VERY LOW	Important

[1] Allocation concealment and attrition bias
[2] Low number of events (<300)
[3] Comparison group had higher values at baseline

d. Identification of pregnancy complications

Quality assessment							No. of participants		Effect		Certainty (GRADE)	Importance
No. of studies	Study design	Risk of bias	Inconsistency	Indirectness	Imprecision	Other considerations	Intervention	Control	Relative (95% CI)	Absolute (95% CI)		
Identification of pregnancy complications<br>Studies: Mori, 2015 (Mongolia)												
1	cRCT	Serious[1]	Not serious	Not serious	Very serious[2]	12 control participants received the intervention	31/252	14/247	OR 2.33 (1.21 to 4.51)	66 more per 1000 (from 11 to 157 more)	VERY LOW	Important

[1] Serious concerns regarding confounding
[2] Very low number of events (<100)

5. For women during pregnancy and after birth, and for caregivers (P), does any use of home-based records (I), compared with inconsistent use (low use) of any home-based records (C), improve health service outcomes (O)?

(no studies)

6. For women during pregnancy and after birth, and for caregivers (P), does use of different types of home-based records (I) and (C) improve health service outcomes (O)?

6.1 Quality of care

a. Communication between women/caregivers and health providers

Quality assessment							No. of participants		Effect		Certainty (GRADE)	Importance
No. of studies	Study design	Risk of bias	Inconsistency	Indirectness	Imprecision	Other considerations	Intervention	Control	Relative (95% CI)	Absolute (95% CI)		
Communication: providers explained everything to them<br>Studies: Homer, 1999 (Australia)												
1	RCT	Serious[1]	Not serious	Not serious	Very serious[2]	None	Not reported	Not reported	Not reported	Statistically significant effect on outcome (P=0.03)	VERY LOW	Important
Communication: records helped talk with doctors<br>Studies: Homer, 1999 (Australia)												
1	RCT	Serious[1]	Not serious	Not serious	Very serious[3]	None	60/65	58/62	OR 0.83 (0.21 to 3.24)	Not calculated	VERY LOW	Important
Communication: easier to talk with doctors<br>Studies: Elbourne, 1987 (UK)												
1	RCT	Serious[4]	Not serious	Not serious	Very serious[5]	None	48/132	25/119	Rate Ratio 1.73 (1.16 to 2.59)<br>OR 2.15 (1.22 to 3.78)	154 more per 1000 (from 35 to 291 more)	VERY LOW	Important

[1] High risk for selection, performance, and attrition bias
[2] Unable to assess number of events as not reported
[3] Small sample size (less than 300 events)
[4] High risk for selection, performance, detection and attrition bias
[5] Small number of events (less than 100)

b. Satisfaction with services

Quality assessment							No. of participants		Effect		Certainty (GRADE)	Importance
No. of studies	Study design	Risk of bias	Inconsistency	Indirectness	Imprecision	Other considerations	Intervention	Control	Relative (95% CI)	Absolute (95% CI)		
Satisfaction with services: satisfaction<br>Studies: Lovell, 1987 (UK); Elbourne, 1987 (UK												
2	RCTs	Very serious[1]	Serious[2]	Serious[3]	Not serious	None	66/95 (Lovell, 1987) No data provided (Elbourne, 1987)	58/102 (Lovell, 1987) No data provided (Elbourne, 1987)	OR 1.73 (0.96 to 3.1) (Lovell, 1987) No data provided (Elbourne, 1987)	Not calculated	VERY LOW	Important
Satisfaction with services: Feeling in control during antenatal care<br>Study: Elbourne, 1987 (UK)												
1	RCT	Serious[1]	Not serious	Not serious	Very serious[4]	None	Enhanced feeling of control: 66/132	Enhanced feeling of control: 41/119	Rate Ratio 1.45 (1.08 to 1.95)	155 more per 1000 (from 28 to 327 more)	VERY LOW	Important
Satisfaction with services: Positive comments included a sense of control<br>Study: Homer, 1999 (Australia)												
1	RCT	Serious[5]	Not serious	Serious[6]	Serious[7]	None	Positive comments, including a sense of control: 58/65	Positive comments, including a sense of control:55/62	OR 1.05 (0.35 to 3.2)	Not calculated	VERY LOW	Important

[1] High risk for selection, performance, detection and attrition bias
[2] More frequently satisfied with aspects of care approaching significance for ability to choose companion during labour (Lovell, 1987))
[3] Population comprised of a higher proportion of one-parent families, high unemployment rate and a quarter of sample included West Indian and other groups disproportionately affected by social-deprivation
[4] Small number of events
[5] High risk for selection, performance and attrition bias
[6] Proxy measure of outcome (indirect evidence)
[7] Small sample size
