# Supplementary material for: Effectiveness of home-based records on maternal, newborn and child health outcomes: A systematic review and meta-analysis
Source: PLoS One. 2019 Jan 2;14(1):e0209278. doi: 10.1371/journal.pone.0209278 (PMC6314587; doi:10.1371/journal.pone.0209278)
Supplement: S4 File — (PDF) [file pone.0209278.s004.pdf]

Effectiveness and cost-effectiveness of home-based records on maternal, newborn and child health outcomes: A protocol for a WHO systematic review and meta-analysis

Olivia Magwood[1,2], Alain Mayhew[1], Victoire Kpade[1], Kednapa Thavorn Sandy Oliver[4], Joanna Vogel[5], Kevin Pottie[1,6]

1. *C.T. Lamont Primary Health Care Research Centre, Bruyère Research Institute, Ottawa ON*
2. *Department of Population Medicine, University of Guelph, Guelph, Canada*
3. *Ottawa Hospital Research Institute, Ottawa, Ontario, Canada*
4. *University College London, London, UK*
5. *Office of the Assistant Director-General, Family, Women's and Children's Health*
6. *Departments of Family Medicine & Epidemiology and Community Medicine, University of Ottawa, Canada*

BACKGROUND

A home-based record (HBR) is a paper or electronic health record retained and used by women or caregivers in the household to document maternal, newborn and child health (MNCH). This document may include components of preventive and/or curative antenatal, postnatal, newborn, and child health, vaccination and nutrition. In 1948, the Japanese Health System introduced a MNCH handbook to improve the health of vulnerable mothers and children (Hagiwara, 2013). Since, at least 163 countries or territories have been known to use some form of home based records (WHO, 2017). However, there is no standardized format of content and design of HBRs across and even within certain countries. This inconsistency can impact health reporting, health outcomes, scaling up, and evaluation between different health systems in the world. The content, design and durability of HBRs are crucial for their effectiveness and their implementation (WHO, 2015).

The use of HBRs may increase continuity of care by containing reminders of following health visits, improving newborn and children adherence to vaccination and monitoring of child development. HBRs may also help women effectively address and prevent complications for themselves and their child by providing health information on preventative measures to maintain a healthy pregnancy. Studies show that involving women in the management of their care decreases anxiety, increases their feeling of empowerment, and improves both their health outcomes and those of their children (Shaw, 2008; Brown, 2004). These records are also likely to improve communication between patients and healthcare providers. If all of the providers are recording in the same record, it would improve provider to provider communication and reduce clinical errors (Brown, 2004).

Studies have analysed the efficacy and effectiveness of mobile and paper-based HBRs for improving health outcomes associated with chronic diseases like cancer or mental health, but very few have systematically assessed the effectiveness and cost-effectiveness of HBRs for improving MNCH nor a comparison of the benefits of using different types of HBRs (Phipps, 2001).

OBJECTIVES

The objective of this project is to synthesize the evidence of the effectiveness and cost-effectiveness of HBRs for women and children, considering maternal, newborn and child health outcomes and empowerment outcomes for women.

METHODS

Part A. Conduct a Systematic Review of RCTs and controlled trials

Key Questions:

Part A

1. For women during pregnancy and after birth (P), does use of HBRs (I) as compared with no HBRs (C), improve maternal health outcomes (O)?
2. For women during pregnancy and after birth (P), does use of a specific type of home-based record (I), compared with use of a different type of home-based record, (C), improve maternal health outcomes (O)?
3. For newborns and children (P), does use of home-based records (I), as compared with no use of any home-based records (C), improve newborn and child health outcomes (O)?
4. For newborns and children (P), does use of a specific type of home-based record (I), compared with use of a different type of home-based record, (C), improve newborn and child health outcomes (O)?
5. For women (P), does use of home-based records (I), as compared with no use of any home-based records (C), improve women's empowerment outcomes (O)?
6. For women (P), does use of a specific type of home-based record (I), compared with use of a different type of home-based record, OR (C), improve women's empowerment outcomes (O)?
7. What are the potential harms (O) to mothers and children (P) associated with the use of HBRs (I) as compared with no use or other types of HBR (C)?

Part A will focus on a quantitative analysis of the available evidence. Eligible studies for this review will include RCTs and controlled trials that meet the criteria outlined below. We anticipate that there will be many controlled non-randomized studies, and excluding them would greatly limit the availability of the evidence. We are limiting the non-randomized designs to those which have a control group either concurrently (non-randomized controlled trials) or are controlled by time (interrupted time series). We have chosen RCTs and controlled trials to test the effect of the intervention by comparing it to a control condition (no intervention or an alternative intervention) to reduce the impact of factors that may vary over time. This will allow us to draw causal conclusions on whether or not the intervention leads to changes in outcomes. We will also identify relevant systematic reviews and will consider the included primary studies of these reviews for eligibility. If more than one version of a study is

identified, we will select the most recent version. If the two versions report on different outcomes, both studies will be included.

Types of participants

Women: Studies that consider any pregnant women regardless of age, health status and number of pregnancies, will be included. Studies that include mothers with children under the age of ten will be included.

Children: Studies which focus on infants less than 1 year of age and children under 10 years of age (WHO, 2013).

Types of interventions

The intervention of interest is any form of a HBR which focuses on either maternal or child health outcomes, or both, which may include but is not limited to: vaccination only records (record of basic identifying information and immunization services received), vaccination-plus records (record of child growth and development, immunization services, and a limited set of basic information related to child survival), child health book (record of birth characteristics, health services received, growth and feeding practices, guidance to parents), pregnancy case-notes, and maternity personal health records. Patient diaries will not be considered as an eligible intervention. These health records may be in paper form or electronic form to be considered for this review. Patient-held records must be a component of the intervention.

Outcomes

Part A of this project will focus on the outcomes listed in Table 1. Other important outcomes will be recorded and analyzed. We will also conduct a subgroup analysis on equity to highlight groups not receiving or not benefiting from HBRs.

Critical maternal health outcomes	Critical newborn and child health outcomes	Secondary Outcomes
**1. Maternal health reporting**<br>• Antenatal care visits<br>• Pregnancy complications<br>• Maternal immunization<br>• Childbirth with skilled birth attendant or at a health facility<br>• Number of postpartum care visits<br><br>**2. Maternal care seeking and self-care practice**<br>• Antenatal care visits<br>• Pregnancy complications<br>• Maternal immunization<br>• Childbirth with skilled birth attendant or at a health facility	**1. Newborn and child health reporting**<br>• Full vaccination series completion<br>• Growth and development monitoring<br>• Care-seeking for childhood illness<br><br>**2. Newborn and child health care-seeking and care practice**<br>• Full vaccination series completion<br>• Growth and development monitoring<br>• Care-seeking for childhood illness<br>• Immediate and continued	**Women's empowerment**<br>• Increased knowledge of maternal and child health<br>• Strengthened communication within the household<br>• Strengthened communication between women and health care provider<br>• Increased agency for women to seek antenatal/maternal care

• Postpartum care visits<br>• Receipt of maternal immunization<br>• Healthy pregnancy nutrition<br>• Postpartum family planning<br>• Healthy household environment<br>**3. Maternal mortality & morbidity**	exclusive breastfeeding<br>• Warmth and hygiene of the newborn<br>• Healthy infant and young child feeding<br>• Illness management<br>**3. Child mortality & morbidity**	

Search Strategy
A librarian will develop and peer-review a search strategy. The following electronic databases will be searched for systematic reviews, RCTs and controlled trials: MEDLINE, CENTRAL, EMBASE, Health Systems Evidence and CINAHL. There will be no date or language restrictions set for the search. The search strategy will use a combination of indexed terms and free text words. In addition we will search grey literature for published guidelines and reports on home-based records on CDC, ECDC, and WHO websites. The literature search results will be uploaded to a reference manager software package to facilitate the study selection process.

Study Screening and Selection
Two review authors will independently assess all the potential studies using inclusion/exclusion criteria. We will resolve any disagreements through discussion or, if required, we will consult a third review author. During the systematic review, relevant citations that are not controlled trials or RCTs will be catalogued so they are available if needed at a later stage. Study selection will be verified on up to 10% of the studies by a member of the funding agency.

Data Extraction
We will develop a standardised extraction sheet. Two reviewers will extract data in duplicate and independently. They will compare results and resolve disagreements by discussion or with help from a third reviewer. Data extraction will be verified on up to 10% of the studies by a member of the funding agency.

Assessment of risk of bias in included studies and certainty of the evidence
Two reviewers will assess the quality of the included RCTs and controlled trials in duplicate and disagreements will be resolved by discussion or using a third reviewer. The quality of randomized trials will be assessed with the risk of bias tools from the Cochrane Handbook. The quality of nonrandomized controlled trials will also be assessed using the Cochrane risk of bias tool, but will receive a judgement of “high risk of bias” for random allocation and allocation concealment by default.

The GRADE criteria will be applied to assess the certainty of evidence for the included studies. The rating is based on an assessment of: 1) risk of bias (study limitation); 2) inconsistency (heterogeneity) in the direction and/or size of the estimates of effect; 3) indirectness of the body of evidence to the populations, interventions, comparisons and/or outcomes; 4)

imprecisions of results (few participant/events/observations and/or wide confidence intervals (CIs)); 5) other considerations (effect size and publication bias) (Phipps, 2001). The certainty of evidence may be downgraded if there are serious or very serious concerns related to any of the GRADE criteria. All key data will be entered in the GRADEpro software. This software will be used to produce GRADE evidence profile tables and summary of findings tables.

Quantitative Analysis

We anticipate multiple and heterogeneous interventions and outcomes. We will use an effect driven plot graph to help identify outliers results and to help determine the best grouping for meta-analysis. We will therefore use a meta-analysis when it is appropriate to statistically synthesise studies. Results will be presented with a 95%CI and estimates of Tau^2 or I^2. When possible to combine studies, dichotomous outcomes will be reported as relative risks and continuous outcomes will be reported as weighted mean differences. If an outcome has been assessed using different scales, standardised mean differences would be used to summarise the data, when possible. When it is not possible to combine the data, the results for each study will be presented separately. If a meta-analysis is not possible, we will narratively synthesise and summarise the data in effect driven plots.

Part B: Conduct a systematic review of economic evaluation studies of use of home-based records

Key Question:

1. Compared with no-use of any home-based records, are single/multi domain home-based record interventions cost effective in LMIC?

Search Strategy

A search strategy will be developed and peer-reviewed by a librarian and a health economist. The following electronic databases will be searched for systematic reviews and RCTs: MEDLINE, CENTRAL, EMBASE, CINAHL, HTA database, NHSEED, and DARE. There will be no date restrictions set for the search. The search strategy will use a combination of indexed terms and free text words.

Several websites of relevant organizations will be searched as sources for grey literature, including the Canadian Agency for Drugs and Technologies in Health, the Institute of Health Economics, the National Institute for Health and Care Excellence, EuroScan, CDC, ECDC, UNAIDS, WHO and the Centre for Reviews and Dissemination database. Similar to Part A, search records will be uploaded to a reference manager software package to facilitate the study selection process.

Inclusion/exclusion Criteria and Study Screening

We will include any primary studies or health technology assessment reports that assessed costs and outcomes of HBRs. These studies may consist of a cost minimization study, a cost-benefit study, a cost-effectiveness study, or a cost-utility study. We will use the same process as Part A to screen titles/abstract and full-text articles.

Data Extraction

We will develop a standardised extraction sheet. Teams of two reviewers will extract data in duplicate and independently. Disagreements will be resolved through consensus or the involvement of a third author. We will abstract study characteristics, including study design, study location (and respective currency), modelling method (e.g. decision tree or Markov), the study's eligible population, and intervention/comparator. The perspective of the economic evaluation will be categorised as: patient, hospital, healthcare system, or society.

Results of economic evaluation studies will be reported as an incremental cost effectiveness ratio (ICER), which is derived by dividing the difference in cost between use of HBRs and the comparator (incremental cost) by the difference in effectiveness of use of HBRs and the comparator (incremental effectiveness). Effectiveness may be reported as life years saved, quality-adjusted life years, or disability-adjusted life years.

Methodological Quality Appraisal

The methodological quality of the included studies will be appraised using the 10-item Drummond checklist (Drummond, 2015). This tool is commonly used and recommended by the Cochrane Collaboration for assessing the quality of health economic evaluation studies. Each study will be appraised independently by two team members. Any conflicts will be resolved through discussion and consensus. A score of eight or higher (out of ten) indicates that the study is methodologically sound and the authors made a concerted effort to describe the nature of the study in rigorous detail.

Synthesis

The results of included studies will be reported narratively. Cost data from each included study was converted to 2017 U.S. Dollars (USD) using purchasing power parity for the year of publication of each study then adjusted for inflation to the year 2017.

DISSEMINATION OF RESULTS

We will publish this systematic review in an open access journal.

FUNDING SOURCES

This work is supported by the Japan International Cooperation Agency (JICA).

CONFLICTS OF INTEREST

There are no potential conflicts of interest to report.

REFERENCES

Brown, H. C., Smith, H. J., Mori, R., & Noma, H. (2015). Giving women their own case notes to carry during pregnancy. *Cochrane Database of Systematic Reviews,* (10), 239-239. doi:10.1002/14651858.CD002856.pub3

Drummond, M. (2015). *Methods for the economic evaluation of health care programmes*. Oxford, United Kingdom: Oxford University Press.

Hagiwara, A., Ueyama, M., Ramlawi, A., & Sawada, Y. (2012). Is the Maternal and Child Health (MCH) handbook effective in improving health-related behavior? Evidence from Palestine. *Journal of Public Health Policy, 34*(1), 31-45. doi:10.1057/jphp.2012.56

Home-based Record Repository. (2017). Retrieved November 23, 2017, from http://www.immunizationcards.org/

Home-based Record Repository. (2017). Retrieved June 23, 2017, from http://www.immunizationcards.org/

Phipps, H. (2001). Carrying their own medical records: the perspective of pregnant women. *The Australian and New Zealand Journal of Obstetrics and Gynaecology, 41*(4), 398-401. doi:10.1111/j.1479-828x.2001.tb01316.x

Shaw, E., Howard, M., Chan, D., Waters, H., Kaczorowski, J., Price, D., & Zazulak, J. (2008). Access to Web-Based Personalized Antenatal Health Records for Pregnant Women: A Randomized Controlled Trial. *Journal of Obstetrics and Gynaecology Canada, 30*(1), 38-43. doi:10.1016/s1701-2163(16)32711-6

World Health Organization. (n.d.). Home-based Record Repository. Retrieved June 23, 2017, from http://www.immunizationcards.org/

World Health Organization. (2013). Definition of key terms. Retrieved June 23, 2017, from http://www.who.int/hiv/pub/guidelines/arv2013/intro/keyterms/en/

World Health Organization. (2015). Practical Guide for the Design, Use and Promotion of Home-Based Records in Immunization. Retrieved June 23, 2017, from http://www.who.int/immunization/monitoring_surveillance/routine/homebasedrecords/en/

World Health Organization. (2017). Home-based Record Repository. Retrieved June 23, 2017, from http://www.immunizationcards.org/
